# Supplementary material for: A nanopore-based HIV-1 reference epitranscriptome
Source: Nucleic Acids Res. 2026 Mar 19;54(6):gkag220. doi: 10.1093/nar/gkag220 (PMC13000461; doi:10.1093/nar/gkag220)
Supplement: gkag220_Supplemental_Files [file gkag220_supplemental_files.zip › Bosmeny_HIV1-Ref-epi_Supplemental_full.pdf]

Supplementary Table S1. DNA oligonucleotides used in this study.

Name	Sequence(5'->3')	Notes
NL4-3_Seg1_F_T7	TAATACGACTCACTATAgggagaagtattagtgtggaag	Generates 3452nt PCR fragment for sense
NL4-3_Seg1_R	GGGTCATAATACACTCCATGTACC	transcription of NL4-3 from 1 to 3048nt.
NL4-3_Seg1_F	gggagaagtattagtgtggaag	Generates 3452nt PCR fragment for antisense
NL4-3_Seg1_R_T7	TAATACGACTCACTATAGGGTCATAATACACTCCATGTACC	transcription of NL4-3 from 1 to 3048nt.
NL4-3_Seg2_F_T7	TAATACGACTCACTATAGGGAAGTTCAATTAGGAATACCAC	Generates 3065nt PCR fragment for sense
NL4-3_Seg2_R	GGGCTCTAGTCTAGGATCTAC	transcription of NL4-3 from 2359 to 5406nt.
NL4-3_Seg2_F	GGGAAGTTCAATTAGGAATACCAC	Generates 3065nt PCR fragment for antisense
NL4-3_Seg2_R_T7	TAATACGACTCACTATAGGGCTCTAGTCTAGGATCTAC	transcription of NL4-3 from 2359 to 5406nt.
NL4-3_Seg3_F_T7	TAATACGACTCACTATAGGGAGTCTCCATAGAATGGAG	Generates 3101nt PCR fragment for sense
NL4-3_Seg3_R	GGGTCTGAAACGATAATGGTG	transcription of NL4-3 from 4836 to nt.
NL4-3_Seg3_F	GGGAGTCTCCATAGAATGGAG	Generates 3101nt PCR fragment for antisense
NL4-3_Seg3_R_T7	TAATACGACTCACTATAGGGTCTGAAACGATAATGGTG	transcription of NL4-3 from 4836 to 7919nt.
NL4-3_Seg4_F_T7	TAATACGACTCACTATAGGGCATCAAACAGCTCC	Generates 1747nt PCR fragment for sense
NL4-3_Seg4_R	gggatctctagttaccagagtc	transcription of NL4-3 from 7474 to 9172nt.
NL4-3_Seg4_F	GGGCATCAAACAGCTCC	Generates 1747nt PCR fragment for antisense
NL4-3_Seg4_R_T7	TAATACGACTCACTATAgggatctctagttaccagagtc	transcription of NL4-3 from 7474 to 9172nt.

NL520R	TAAGCAGTGGGTTCCCTAGT	Primers from Baek et al. (2024)
NL590R	GAGGGATCTCTAGTTACCAG	for improved RT during sequencing prep
NL660R	CTTTCAAGTCCCTGTTCGGG	
NL780R	TCTAGCCTCCGCTAGTCAAA	
NL870R	TTTCTTTCCCCCTGGCCTTA	
NL930R	AAAGGCCAGGATTAACTGCG	
NL1010R	TCTTCTGATCCTGTCTGAAG	
NL1097R	TTATCTAAGGCTTCCTTGGTGTC	
NL1160R	TTTCCTGTGTCAGCTGCTGC	
NL1240R	TTCTAGGTGATATGGCCTGA	
NL1290R	TTCTGGGCTGAAAGCCTTCT	
NL1380R	GGCTGCTTGATGTCCCCCCA	
NL1460R	GCAATAGGCCCTGCATGCAC	
NL1550R	GGATTATGTGTCATCCATCC	
NL1640R	TCCAGAATGCTGGTAGGGCT	
NL1720R	GTGAAGCTTGCTCGGCTCTT	
NL1820R	TCTAGTGTCGCTCCTGGTCC	
NL1910R	GCTGGATTTGTTACTTGGCT	
NL2010R	GGCCCTGCAATTTTTGGCTA	
NL2120R	CTTCCCTTGTGGGAAGGCCA	
NL2210R	TGAGAGGGAGTTGTTGTCTC	
NL2280R	GGGGTCGCTGCCAAAGAGTG	
NL2370R	CTGGCAAATTCATTTCTTCT	
NL2470R	CCTATAGCTTTATGTCCGCA	
NL2550R	GAAAATTTAAAGTGCAGCCA	
NL2610R	CTTTTGGGCCATCCATTCCT	
NL2710R	ATTGTATGGATTTTCAGGCC	
NL2800R	GAACTTCCCAGAAATCTTGA	
NL2890R	GCATCGCCCACATCCAGTAC	
NL2980R	TATCTAATCCCTGGTGTCTC	
NL3090R	AGATGACTATGTCTGGATTT	
NL3180R	TCAACAGATGTTGTCTCAGT	
NL3270R	CTGTCCATTTATCAGGATGG	
NL3350R	TGCCCAATTCAATTTTCCCA	
NL3420R	CTGTTAGTGCTTTGGTTCCC	
NL3500R	CACTCCATGTACCGGTTCTTTTAG	
NL3570R	TTTGATATGTCCATTGGCCT	

NL3660R	CCTCTGTTAATTGTTTCACA
NL3760R	TCTGTCCACCATGCTTCCCA
NL3840R	GTACCATAACTTCACTAAGG
NL3890R	ATTGGCTGCCCCATCTACAT
NL3940R	CCGTTAGGGGGACAACTTTT
NL4060R	CCCAATGCATATTGTGAGTC
NL4170R	CTTTGTGTGCTGGTACCCAT
NL4250R	CTTATCTATTCCATCTAAAA
NL4320R	CAGGTGGTAGGTTAAAATCA
NL4400R	GCTACAGTCTACTTGTCCAT
NL4500R	CTGGAATTACTTCTGCTTCT
NL4600R	GTACTGGTGAAATTGCTGCC
NL4680R	TTACTCCTTGACTTTGGGGATTG
NL4760R	TTGTACTGCTGTCTTAAGAT
NL4850R	GTCTGTTGCTATTATGTCTA
NL4920R	TGCTGTCCCTGTAATAAACC
NL4990R	CTCATCCTGTCTACTTGCCA
NL5030R	GATCTTTGCTTTTCTTCTTGGCACTACT
NL5180R	GTACTTTCATAGTGATGTCT
NL5270R	CTTTCTCCTGTATGCAGACC
NL5360R	ATTAGTTGGTCTGCTAGGTC
NL5440R	CACACCTAGGACTAACTATA
NL5540R	CTAACACTAGGCAAAGGTGG
NL5610R	ATTCATTGTATGGCTCCCTCTG
NL5720R	CCAAGTATCCCCGTAAGTTT
NL5820R	TCCTCTGTCGAGTAACGCCT
NL5880R	CTGACTTCCTGGATGCTTCC
NL5940R	ACAAACTTGGCAATGAAAGC
NL6020R	GTCTGACTGTTCTGATGAGC
NL6130R	ATGATTACTATGGACCACAC
NL6214R	TGTCTTCTGCTCTTTCTATTAGTCTA
NL6300R	ATCATCAATATCCCAAGGAG
NL6400R	TGCTTTAGCATCTGATGCAC
NL6510R	TTCCACATGTTAAAATTTTCTGTCACATT
NL6590R	GGGTTAATTTTACACATGGC
NL6660R	ATTCTCCCGCTACTACTATT
NL6740R	CATATTCTTTCTGCACCTTA
NL6800R	AACTTATCAACCTATAGCTG
NL6880R	ACCAGCCGGGGCACAATAAT
NL6950R	GTACTGTGCTGACATTTGTA
NL7020R	TCTTCTTCTGCTAGACTGCC
NL7112R	TGGGTCTTGTACAATTAATTTCTACAGA
NL7200R	GCTTGTCTCATATTTCCTAT
NL7310R	CTGAGGATTGCTTAAAGATT
NL7370R	TACAGTAGAAAAATTCCCCT
NL7440R	TCAGTGTTATTTGACCCTTC
NL7500R	TCCTGCCACATGTTTATAAA
NL7580R	TTGTTAATAGCAGCCCAGTA
NL7660R	TTCACTTCTCCAATTGTCCC
NL7740R	TCTCTCTGCACCACTCTTCT
NL7820R	TCATTGACGCTGCGCCCATA
NL7910R	ACAGATGCTGTTGCGCCTCA
NL7970R	ATCTTTCCACAGCCAGGATT
NL8040R	GGCACAGCAGTGGTGCAAAT
NL8120R	TAATTTCTCTGTCCCACTCC
NL8180R	TCTTTTCTTGCTGGTTTTGC
NL8380R	TGGGAGGTGGGTCTGAAACG

NL8450R	TGGATCTGTCTCTGTCTCTC	
NL8520R	TGGTAGCTGAAGAGGCACAG	
NL8600R	AATATTTGAGGGCTTCCCAC	
NL8680R	TACTGCTATGGCTGTGGCAT	
NL8750R	TTCTAGGTATGTGGCGAATA	
NL8810R	ACTTTTTGACCACTTGCCAC	
NL8894R	TACTTGTGATTGCTCCATGTTTTTCTAGG	
NL8980R	TCTTCCTCCTCTTGTGCTTC	
NL9050R	GCTAAGATCTACAGCTGCCT	
NL9140R	TTGTGTGTGGTAGATCCACA	
NL9220R	AGCTTGTAGCACCATCCAAA	
NL9290R	CACAGGGTGTAACAAGCTGG	
NL9360R	GAAATGCTAGGCGGCTGTCA	
NL9530R	CCAGTACAGGCAAAAAGCAG	
NL9600R	AGGCTTAAGCAGTGGGTTCC	
NL9660R	ACCAGAGTCACACAACAGAC	

AG9454_28_90	CCGCCCCCTCCGGGGAGGAGGAGGAGGGGCGGCGGGGGAAGGGAGGGCGG	Oligomers used in enzymatic depletion of ribosomal RNA
AG9405_28_41	GGGCGCGGGGTGGGGAGGGAGCGAGCGGCGCGCGGGTGGGGCGGGGGA	
AG9443_28_79	GCACCCCCCCCGTCGCCGGGGCGGGGGCGCGGGGAGGAGGGGTGGGAGAG	
AG9449_28_85	CCCGACCCGCGCGCCCTCCCGAGGGAGGACGCGGGGCCGGGGGGCGGAGA	
AG9421_28_57	GGGCGGGGAGCGGGGCGTGGGCGGGAGGAGGGGAGGAGGCGTGGG	
AG9447_28_83	GGGATTCGGCGAGTGCTGCTGCCGGGGGGGCTGTAACACTCGGGGIGGGT	
AG9396_28_32	GCCGCCGCCGCCGCCGCGCGCCGAGGAGGAGGGGGGAACGGGGGGCGGAC	
AG9404_28_40	CTCCCCCGGGGAGGGGGGAGGACGGGGAGCGGGGGAGAGAGAGAGAGAGA	
AG9514_12_15	CGCGGTGGCTGGCACGAAATTGACCAACCCTGGGGTTAGTATAGCTTAGT	
AG9349_18_23	TAAGAGCATCGAGGGGGCGCCGAGAGGCAAGGGGCGGGGACGGGCGGTGG	
AG9398_28_34	CCGACCGCCGCCGCCCGACCGCTCCCGCCCCCAGCGGACGCGCGCGCGAC	
AG9370_28_6	GGGCGCGTGGAGGGGIGGGCGGCCCGCCGGCGGGGACAGGCGGGGGACCG	
AG9373_28_9	TTCGCCCCATTGGCTCCTCAGCCAAGCACATACACCAAATGTCTGAACCT	
AG9452_28_88	GGTGGAAATGCGCCCGGCGGCGGCCGGTCGCCGGTCGGGGGACGGTCCCC	
AG9346_18_20	CTTAATCATGGCCTCAGTTCCGAAAACCAACAAAATAGAACCGCGGTCCT	
AG9430_28_66	CCATTTAAAGTTTGAGAATAGGTTGAGATCGTTTCGGCCCCAAGACCTCT	
AG9395_28_31	CCGGGGGCGGACCCGGCGGGIGGGACCGGCCCGCGGCCCCTCCGCCGCCT	
AG9450_28_86	CGGGGGAGGAGGAGGACGGACGGACGGACGGGGCCCCCCGAGCCACCTTC	
AG9501_12_2	CTCTATATAAATGCGTAGGGGTTTTAGTTAAATGTCCTTTGAAGTATACT	
AG9335_18_9	GGTGGCTGAACGCCACTTGTCCCTCTAAGAAGTTGGGGGACGCCGACCGC	
AG9482_16_14	GGGTACCGCGGCCGTTAAACATGTGTCACTGGGCAGGCGGTGCCTCTAAT	
AG9406_28_42	GGGCCGCGAGGGGGGTGCCCCGGGCGTGGGGIGGGCGCGCGCCTCGTCCA	
AG9399_28_35	CGAGACGTGGGGTGGGGGTGGGGGGCGCGCCGCGCCGCCGCCGGGCTCCC	
AG9365_28_1	GACAAACCCTTGTGTCGAGGGCTGACTTTCAATAGATCGCAGCGAGGGAG	
AG9361_18_35	TTAGCTCTAGAATTACCACAGTTATCCAAGTAGGAGAGGAGCGAGCGACC	
AG9389_28_25	TGGATAGTAGGTAGGGACAGTGGGAATCTCGTTCATCCATTCATGCGCGT	
AG9384_28_20	CCCTCGGGCTCGCCCCCCCGCCTCACCGGGTCAGTGAAAAAACGATCAGA	
AG9372_28_8	GGCGGGATTCTGACTTAGAGGCGTTCAGTCATAATCCCACAGATGGTAGC	
AG9383_28_19	GGAGCGGGTCGCGCCCGGCCGGGCGGGCGCTTGGCGCCAGAAGCGAGAGC	
AG9333_18_7	CCTGCCGGCGTAGGGTAGGCACACGCTGAGCCAGTCAGTGTAGCGCGCGT	
AG9444_28_80	CGGTCGCGCCGTGGGAGGGGTGGCCCGGCCCCCCCACGAGGAGACGCCGG	
AG9350_18_24	CTCGCCTCGCGGCGGACCGCCCGCCCGCTCCCAAGATCCAACTACGAGCT	
AG9445_28_81	CGCGCCCCCGCGGGGGAGACCCCCCTCGCGGGGGATTCCCCGCGGGGGTG	
AG9341_18_15	CTCCCCCCGGAACCCAAAGACTTTGGTTTCCCGGAAGCTGCCCGGCGGGT	
AG9456_28_92	ACACGGCCGGACCCGCCGCCGGGTTGAATCCTCCGGGCGGACTGCGCGGA	
AG9441_28_77	GACGCGCGCGTGGCCCCGAGAGAACCTCCCCCGGGCCCGACGGCGCGACC	
AG9327_18_1	TAATGATCCTTCCGCAGGTTCACCTACGGAAACCTTGTTACGACTTTTAC	
AG9344_18_18	ATGAAAACATTCTTGGCAAATGCTTTCGCTCTGGTCCGTCTTGCGCCGGT	
AG9400_28_36	CGGGGGCGGCCGCGACGCCCGCCGCAGCTGGGGCGATCCACGGGAAGGGC	
AG9512_12_13	GAGTTTTTTACAACTCAGGTGAGTTTTAGCTTTATTGGGGAGGGGGTGAT	

AG9397_28_33	GGGCCGGGIGGGTAGGGCGGGGGACGAACCGCCCCGCCCCGCCGCCCG
AG9401_28_37	CCGGCTCGCGTCCAGAGTCCGCGCCGCCGCCGGCCCCCCGGGTCCCCGGG
AG9359_18_33	GCCCCCCGGCCGGGGCCGGAGAGGGGCTGACCGGGTTGGTTTTGATCTGA
AG9431_28_67	AATCATTCGCTTTACCGGATAAAACTGCGTGGCGGGGGTGCGTCGGGTCT
AG9466_5.8_1	AAGCGACGCTCAGACAGGCGTAGCCCCGGGAGGAACCCGGGGCCGCAAGT
AG9422_28_58	GGGCGGGGGAAGGACCCCACACCCCCGCCGCCGCCGCCGCCGCCGCCCTC
AG9394_28_30	AGTCGGCTGCTAGGCGCCGGCCGAGGCGAGGCGCGCGCGGAACCGCGGCC
AG9469_16_1	AAACCCTGTTCTTGGGTGGGTGTGGGTATAATACTAAGTTGAGATGATAT
AG9439_28_75	ACGTGTTAGACTCCTTGGTCCGTGTTTCAAGACGGGTCGGGTGGGTAGCC
AG9436_28_72	CTCCACCTCCCCGGCGCGGCGGGCGAGACGGGCCGGTGGTGCGCCCTCGG
AG9499_16_31	AATGGTTTGGCTAAGGTTGTCTGGTAGTAAGGTGGAGTGGGTTTGGGGCT
AG9461_28_97	GCCTCACACCGTCCACGGGCTGGGCCTCGATCAGAAGGACTTGGGCCCCC
AG9446_28_82	GGCGCCGGGAGGGGGGAGAGCGCGGCGACGGGTCTCGCTCCCTCGGCCCC
AG9329_18_3	GTGGGCCGACCCCGGCGGGGCCGATCCGAGGGCCTCACTAAACCATCCAA
AG9357_18_31	TCGAAAGTTGATAGGGCAGACGTTCGAATGGGTCGTCGCCGCCACGGG
AG9352_18_26	GCTGCTGGCACCAGACTTGCCCTCCAATGGATCCTCGTTAAAGGATTTAA
AG9455_28_91	GTGGAGGGGTCGGGAGGAACGGGGGGCGGGAAAGATCCGCCGGGCCGCCG
AG9374_28_10	GCGGTTCCTCTCGTACTGAGCAGGATTACCATGGCAACAACACATCATCA
AG9386_28_22	CCCTCGCGGGGACACCGGGIGGGCGCCGGGGGCCTCCCACTTATTCTACA
AG9448_28_84	TTCGGTCCCGCCGCCCCCGCCGCCGCCGCCACCGCCGCCGCCGCCGCCGC
AG9418_28_54	ACTTCGGCCTTCAAAGTTCTCGTTTGAATATTTGCTACTACCACCAAGAT
AG9432_28_68	GCGAGAGCGCCAGCTATCCTGAGGGAAACTTCGGAGGGAACCAGCTACTA
AG9518_12_19	CTTGCATGTGTAATCTTACTAAGAGCTAATAGAAAGGCTAGGACCAAACC
AG9351_18_25	TTTTAACTGCAGCAACTTTAATATACGCTATTGGAGCTGGAATTACCGCG
AG9509_12_10	GGCTCGTAGTGTTCTGGCGAGCAGTTTTGTTGATTTAACTGTTGAGGTTT
AG9336_18_10	TCGGGGGTCGCGTAACTAGTTAGCATGCCAGAGTCTCGTTCGTTATCGGA
AG9367_28_3	GGTTTAGCGCCAGGTTCCCCACGAACGTGCGGTGCGTGACGGGCGAGGG
AG9328_18_2	TTCCTCTAGATAGTCAAGTTCGACCGTCTTCTCAGCGCTCCGCCAGGGCC
AG9437_28_73	CGGACTGGAGAGGCCTCGGGATCCCACCTCGGCCGGCGAGCGCGCCGGCC
AG9381_28_17	ACGGGAGGTTTCTGTCCTCCCTGAGCTCGCCTTAGGACACCTGCGTTACC
AG9345_18_19	CCAAGAATTTCACCTCTAGCGGCGCAATACGAATGCCCCCGGCCGTCCCT
AG9348_18_22	CACTCTAATTTTTTCAAAGTAAACGCTTCGGGCCCCGCGGGACACTCAGC
AG9471_16_3	TTCGTACAGGGAGGAATTTGAANGTAGATAGAAACCGACCTGGATTACTC
AG9385_28_21	GTAGTGGTATTTCACCGGCGGCCCGCAGGGCCGCGGACCCCGCCCCGGGC
AG9438_28_74	TTCACCTTCATTGCGCCACGGCGGCTTTCGTGCGAGCCCCCGACTCGCGC
AG9470_16_2	CATTTACGGGGGAAGGCGCTTTGTGAAGTAGGCCTTATTTCTCTTGTCCT
AG9363_18_37	CCGTGCGTACTTAGACATGCATGGCTTAATCTTTGAGACAAGCATATGCT
AG9521_5_3	TTCCGAGATCAGACGAGATCGGGCGCGTTCAGGGTGGTATGGCCGTAGAC
AG9493_16_25	TCTTGGACAACCAGCTATCACCAGGCTCGGTAGGTTTGTCGCCTCTACCT
AG9355_18_29	CCTTCCTTGGATGTGGTAGCCGTTTCTCAGGCTCCCTCTCCGGAATCGAA
AG9520_5_2	CCAGGCCCGACCCTGCTTAGCTTCCGAGATCAGACGAGATCGGGCGCGTT
AG9382_28_18	GTTTGACAGGTGTACCGCCCCAGTCAAACTCCCCACCTGGCACTGTCCCC
AG9358_18_32	GCGTGCGATCGGCCCGAGGTTATCTAGAGTCACCAAAGCCGCCGGCGCCC
AG9362_18_36	AAAGGAACCATAACTGATTTAATGAGCCATTCGCAGTTTCACTGTACCGG
AG9413_28_49	CTGCCCTTCACAAAGAAAAGAGAACTCTCCCCGGGGCTCCCGCCGGCTTC
AG9451_28_87	CCCGCCGGGCCTTCCCAGCCGTCCCGGAGCCGGTCGCGGCGCACCGCCGC
AG9337_18_11	ATTAACCAGACAAATCGCTCCACCAACTAAGAACGGCCATGCACCACCAC
AG9407_28_43	GCCGCGGCGCGCGCCCAGCCCCGCTTCGCGCCCCAGCCCGACCGACCCAG
AG9506_12_7	CTTACTTTGTAGCCTTCATCAGGGTTTGCTGAAGATGGCGGTATATAGGC
AG9425_28_61	ATCCATTTTCAGGGCTAGTTGATTCGGCAGGTGAGTTGTTACACACTCCT
AG9502_12_3	TGAGGAGGGTGACGGGCGGTGTGTACGCGCTTCAGGGCCCTGTTCAACTA
AG9360_18_34	TAAATGCACGCATCCCCCCCGCGAAGGGGGTCAGCGCCCGTCGGCATGTA
AG9420_28_56	ACCGCAGCGGCCCTCCTACTCGTCGCGGCGTAGCGTCCGCGGGGCTCCGG
AG9460_28_96	CCCAAGCAACCCGACTCCGGGAAGACCCGGGCGCGCGCCGGCCGCTACCG
AG9368_28_4	GCGGCCGCCTTTCCGGCCGCGCCCCGTTTCCCAGGACGAAGGGCACTCCG
AG9489_16_21	TTTAGGTAGTGGGTGTTGAGCTTGAACGCTTTCTTAATTGGTGGCTGCTT
AG9402_28_38	GCCCCCCTCGCGGGGACCTGCCCCCGCCGGCCGCCCCGGCGGCCGCCGCG

AG9486_16_18	GGCTGTTAATTGTCAGTTCAGTGTTTTAATCTGACGCAGGCTTATGCGGA
AG9364_18_38	TGGCTTAATCTTTGAGACAAGCATATGCTACTGGCAGGATCAACCAGGTA
AG9513_12_14	CTAAAACACTCTTTACGCCGGCTTCTATTGACTTGGGTTAATCGTGTGAC
AG9377_28_13	ATAGGAAGAGCCGACATCGAAGGATCAAAAAGCGACGTCGCTATGAACGC
AG9412_28_48	CTTTCCAAGGCACGGGCCCCTCTCTCGGGGCGAACCCATTCCAGGGCGCC
AG9440_28_76	GACGTCGCCGCCGACCCCGTGCGCTCGCTCCGCCGTCCCCCTCTTCGGG
AG9490_16_22	TTAGGCCTACTATGGGTGTTAAATTTTTTACTCTCTCTACAAGGTTTTTT
AG9508_12_9	TCCTCTAGAGGGATATGAAGCACCGCCAGGTCCTTTGAGTTTTAAGCTGT
AG9409_28_45	CCCTTACCTACATTGTTCCAACATGCCAGAGGCTGTTCACCTTGGAGACC
AG9414_28_50	TCCGGGATCGGTCGCGTTACCGCACTGGACGCCTCGCGGCGCCCATCTCC
AG9462_28_98	CACGAGCGGCGCCGGGGAGCGGGTCTTCCGTACGCCACATGTCCCGCGCC
AG9330_18_4	TCGGTAGTAGCGACGGGCGGTGTGTACAAAGGGCAGGGACTTAATCAACG
AG9487_16_19	GGAGAATGTTTTCATGTTACTTATACTAACATTAGTTCTTCTATAGGGTG
AG9428_28_64	GTTCGGTTCATCCCGCAGCGCCAGTTCTGCTTACCAAAAGTGGCCCACTA
AG9498_16_30	TATCTATTGCGCCAGGTTTCAATTTCTATCGCCTATACTTTATTTGGGTA
AG9366_28_2	CTGCTCTGCTACGTACGAAACCCCGACCCAGAAGCAGGTCGTCTACGAAT
AG9332_18_6	ATTGCAATCCCCGATCCCCATCACGAATGGGGTTCAACGGGTTACCCGCG
AG9356_18_30	CCCTGATTCCCCGTCACCCGTGGTCACCATGGTAGGCACGGCGACTACCA
AG9459_28_95	TGCCGGTATTTAGCCTTAGATGGAGTTTACCACCCGCTTTGGGCTGCATT
AG9479_16_11	AGGGTCTTCTCGTCTTGCTGTGTTATGCCCGCCTCTTCACGGGCAGGTCA
AG9480_16_12	ATTTCACTGGTTAAAAGTAAGAGACAGCTGAACCCTCGTGGAGCCATTCA
AG9503_12_4	AGCACTCTACTCTTAGTTTACTGCTAAATCCACCTTCGACCCTTAAGTTT
AG9464_28_100	CTGAGGGAATCCTGGTTAGTTTCTTTTCCTCCGCTGACTAATATGCTTAA
AG9457_28_93	CCCCACCCGTTTACCTCTTAACGGTTTCACGCCCTCTTGAACTCTCTCTT
AG9475_16_7	GGGTAACTTGTTCCGTTGGTCAAGTTATTGGATCAATTGAGTATAGTAGT
AG9369_28_5	CACCGGACCCCGGTCCCGGCGCGCGGCGGGGCACGCGCCCTCCCGCGGCG
AG9434_28_70	GCACGTCAGGACCGCTACGGACCTCCACCAGAGTTTCCTCTGGCTTCGCC
AG9408_28_44	CCCTTAGAGCCAATCCTTATCCCGAAGTTACGGATCCGGCTTGCCGACTT
AG9416_28_52	GGCAACGGAGGCCATCGCCCGTCCCTTCGGAACGGCGCTCGCCCATCTCT
AG9507_12_8	TGAGCAAGAGGTGGTGAGGTTGATCGGGGTTTATCGATTACAGAACAGGC
AG9354_18_28	TTTCGTCACTACCTCCCCGGGTCGGGAGTGGGTAATTTGCGCGCCTGCTG
AG9353_18_27	AGTGGACTCATTCCAATTACAGGGCCTCGAAAGAGTCCTGTATTGTTATT
AG9392_28_28	GCACTGGGCAGAAATCACATCGCGTCAACACCCGCCGCGGGCCTTCGCGA
AG9517_12_18	TTCCTTTTGATCGTGGTGATTTAGAGGGTGAACTCACTGGAACGGGGATG
AG9415_28_51	GCCACTCCGGATTCGGGGATCTGAACCCGACTCCCTTTCGATCGGCCGAG
AG9424_28_60	CACTCTCGACTGCCGGCGACGGCCGGGTATGGGCCCGACGCTCCAGCGCC
AG9343_18_17	CTACGACGGTATCTGATCGTCTTCGAACCTCCGACTTTCGTTCTTGATTA
AG9334_18_8	GCAGCCCCGGACATCTAAGGGCATCACAGACCTGTTATTGCTCAATCTCG
AG9467_5.8_2	GCGTTCGAAGTGTCGATGATCAATGTGTCCTGCAATTCACATTAATTCTC
AG9433_28_69	GATGGTTCGATTAGTCTTTCGCCCCTATACCCAGGTCGGACGACCGATTT
AG9391_28_27	TCCCGCCGTTTACCCGCGCTTCATTGAATTTCTTCACTTTGACATTCAGA
AG9478_16_10	GACCTGTGGGTTTGTTAGGTACTGTTTGCATTAATAAATTAAAGCTCCAT
AG9339_18_13	GCCGGGTGAGGTTTCCCGTGTTGAGTCAAATTAAGCCGCAGGCTCCACTC
AG9496_16_28	CAAAGTTATTTCTAGTTAATTCATTATGCAGAAGGTATAGGGGTTAGTCC
AG9515_12_16	TAAACTTTCGTTTATTGCTAAAGGTTAATCACTGCTGTTTCCCGTGGG
AG9474_16_6	AACCCTATTGTTGATATGGACTCTAGAATAGGATTGCGCTGTTATCCCTA
AG9393_28_29	TGCTTTGTTTTAATTAAACAGTCGGATTCCCCTGGTCCGCACCAGTTCTA
AG9387_28_23	CCTCTCATGTCTCTTCACCGTGCCAGACTAGAGTCAAGCTCAACAGGGTC
AG9417_28_53	CAGGACCGACTGACCCATGTTCAACTGCTGTTCACATGGAACCCTTCTCC
AG9331_18_5	CAAGCTTATGACCCGCACTTACTCGGGAATTCCCTCGTTCATGGGGAATA
AG9468_5.8_3	GCAGCTAGCTGCGTTCTTCATCGACGCACGAGCCGAGTGATCCACCGCTA
AG9427_28_63	CACCTTTTCTGGGGTCTGATGAGCGTCGGCATCGGGCGCCTTAACCCGGC
AG9342_18_16	CATGGGAATAACGCCGCCGCATCGCCGGTCGGCATCGTTTATGGTCGGAA
AG9426_28_62	TAGCGGATTCCGACTTCCATGGCCACCGTCCTGCTGTCTATATCAACCAA
AG9376_28_12	CCTATTAGTGGGTGAACAATCCAACGCTTGGCGAATTCTGCTTCACAATG
AG9442_28_78	CGCCCGGGGCGCACTGGGGACAGTCCGCCCCGCCCCCCGACCCGCGCGCG
AG9472_16_4	CGGTCTGAACTCAGATCACGTAGGACTTTAATCGTTGAACAAACGAACCT

AG9519_5_1	AAAGCCTACAGCACCCGGTATTCCCAGGCGGTCTCCCATCCAAGTACTAA	
AG9488_16_20	ATAGATTGGTCCAATTGGGTGTGAGGAGTTCAGTTATATGTTTGGGATTT	
AG9379_28_15	GCTTAAAACCCAAAAGGTCAGAAGGATCGTGAGGCCCCGCTTTCACGGTC	
AG9411_28_47	GATTTTCAAGGGCCAGCGAGAGCTCACCGGACGCCGCCGGAACCGCGACG	
AG9511_12_12	TGTGTTCAGATATGTTAAAGCCACTTTCGTAGTCTATTTTGTGTCAACTG	
AG9371_28_7	GCTATCCGAGGCCAACCGAGGCTCCGCGGCGCTGCCGTATCGTTCGCCTG	
AG9491_16_23	CCTAGTGTCCAAAGAGCTGTTCCTCTTTGGACTAACAGTTAAATTTACAA	
AG9485_16_17	TGGGTTGACAGTGAGGGTAATAATGACTTGTTGGTTGATTGTAGATATTG	
AG9375_28_11	GTAGGGTAAAACTAACCTGTCTCACGACGGTCTAAACCCAGCTCACGTTC	
AG9423_28_59	CGACGCACACCACACGCGCGCGCGCGCGCCGCCCCCGCCGCTCCCGTC	
AG9505_12_6	CCACCTCATGGGCTACACCTTGACCTAACGTCTTTACGTGGGTACTTGCG	
AG9510_12_11	AGGGCTAAGCATAGTGGGGTATCTAATCCCAGTTTGGGTCTTAGCTATTG	
AG9347_18_21	ATTCCATTATTCCTAGCTGCGGTATCCAGGCGGCTCGGGCCTGCTTTGAA	
AG9378_28_14	TTGGCCGCCACAAGCCAGTTATCCCTGTGGTAACTTTTCTGACACCTCCT	
AG9429_28_65	GGCACTCGCATTCCACGCCCGGCTCCACGCCAGCGAGCCGGGCTTCTTAC	
AG9494_16_26	ATAAATCTTCCCACTATTTTGCTACATAGACGGGTGTGCTCTTTTAGCTG	
AG9338_18_12	CCACGGAATCGAGAAAGAGCTATCAATCTGTCAATCCTGTCCGTGTCCGG	
AG9453_28_89	CGCCGACCCCACCCCCGGCCCCGCCCGCCCACCCCCGCACCCGCCGGAGC	
AG9500_12_1	GTTCGTCCAAGTGCACTTTCCAGTACACTTACCATGTTACGACTTGTCTC	
AG9388_28_24	TTCTTTCCCCGCTGATTCCGCCAAGCCCGTTCCCTTGGCTGTGGTTTCGC	
AG9465_28_101	GACTAATATGCTTAAATTCAGCGGGTCGCCACGTCTGATCTGAGGTCGCG	
AG9481_16_13	TACAGGTCCCTATTTAAGGAACAAGTGATTATGCTACCTTTGCACGGTTA	
AG9476_16_8	TCGCTTTGACTGGTGAAGTCTTAGCATGTACTGCTCGGAGGTTGGGTTCT	
AG9390_28_26	CACTAATTAGATGACGAGGCATTTGGCTACCTTAAGAGAGTCATAGTTAC	
AG9410_28_46	TGCTGCGGATATGGGTACGGCCCGGCGCGAGATTTACACCCTCTCCCCCG	
AG9403_28_39	CGGCCCCTGCCGCCCCGACCCTTCTCCCCCCGCCGCGCCCCCACGCGGCG	
AG9435_28_71	CTGCCCAGGCATAGTTCACCATCTTTCGGGTCCTAACACGTGCGCTCGTG	
AG9516_12_17	TGTGGCTAGGCTAAGCGTTTTGAGCTGCATTGCTGCGTGCTTGATGCTTG	
AG9473_16_5	TTAATAGCGGCTGCACCATCGGGATGTCCTGATCCAACATCGAGGTCGTA	
AG9340_18_14	CTGGTGGTGCCCTTCCGTCAATTCCTTTAAGTTTCAGCTTTGCAACCATA	
AG9477_16_9	GCTCCGAGGTCGCCCCAACCGAAATTTTTAATGCAGGTTTGGTAGTTTAG	
AG9504_12_5	CATAAGGGCTATCGTAGTTTTCTGGGGTAGAAAATGTAGCCCATTTCTTG	
AG9483_16_15	ACTGGTGATGCTAGAGGTGATGTTTTTGGTAAACAGGCGGGGTAAGATTT	
AG9492_16_24	GGGATTTAGAGGGTTCTGTGGGCAAATTTAAAGTTGAACTAAGATTCTA	
AG9458_28_94	CAAAGTTCTTTTCAACTTTCCCTTACGGTACTTGTTGACTATCGGTCTCG	
AG9463_28_99	CCGCGGGGCGGGGATTCGGCGCTGGGCTCTTCCCTGTTCACTCGCCGTTA	
AG9380_28_16	TGTATTCGTACTGAAAATCAAGATCAAGCGAGCTTTTGCCCTTCTGCTCC	
AG9495_16_27	TTCTTAGGTAGCTCGTCTGGTTTCGGGGGTCTTAGCTTTGGCTCTCCTTG	
AG9497_16_29	TTGCTATATTATGCTTGGTTATAATTTTTCATCTTTCCCTTGCGGTACTA	
AG9484_16_16	GCCGAGTTCCTTTTACTTTTTTTAACCTTTCCTTATGAGCATGCCTGTGT	
AG9419_28_55	CTGCACCTGCGGCGGCTCCACCCGGGCCCGCGCCCTAGGCTTCAAGGCTC	

2OM_r2r_1087	rUrArCrCrCrUrUrCrArGrGrArArCrArArArUrArGrGrArUrGrGrArUrGmArCrArCrArUrArArUrCrCrArCrCrU rArUrCrCrCrArGrUrArGrGrArGrA	Oligonucleotides synthesized to test Nanopore 2'-O-Me calling.
2OM_r2r_1424	rGrGrGrArCrCrCrGrGrCrCrArUrArArArGrCrArArGrArGrUrUrUmUrGrGrCrUrGrArArGrCrArArUrGrArGrC rCrArArGrUrArArCrArArArU	
2OM_r2r_8404_8408	rCrUrGrUrArArGrGrGrArArArGrArArUrGrArGrArCrGrArGrCrUrGmArGrCrCmArGrCrArGrCrArGrArUrGrG rGrGrUrGrGrGrArGrCrArGrUrArUrC	
2OM_r2r_8960	rCrGrGrArGrUrArCrUrUrCrArArGrArArCrUrGrCrUrGrArCrArUrCrGmArGrCrUrUrGrCrUrArCrArArGrGrG rArCrUrUrUrCrCrGrCrUrGrGrG	

Supplementary Table S2. Significant m6A modification sites in the HIV-1 sense epitranscriptome.

		Jurkat cells with HIV-GFPΔEnv/VSV-G					Jurkat cells with HIV-GFPΔEnv/VSV-G and cART					Jurkats cells (ribosome depletion method)		CD4 T-cells with NL4-3 HIV	CD4 supernatant with NL4-3 HIV	Patient CD4 T-cells			m6A Chemical Knockdown			
Base	T7 InVitro	7C	11E	11B/H	HIV average	HIV stdev	7E	11F	11C/I	HIV+cART average	HIV+cART stdev	Poly-A-selection	RNaseH reaction	10D minus T7	11H minus T7	P5S2 minus T7	P6S minus T7	P8 minus T7	STM2457 0 μM minus T7	STM2457 7.5 μM minus T7	STM2457 30 μM minus T7	STM2457 60 μM minus T7
**125**	0.31%	3.28%	13.97%	16.47%	11.24%	±5.72%	4.27%	9.96%	20.94%	11.73%	±6.92%	17.31%	1.13%	0.44%	0.00%	-	-	-	6.79%	1.53%	1.41%	1.99%
**7983**	0.11%	15.06%	16.78%	16.85%	16.23%	±0.83%	16.52%	14.58%	16.30%	15.80%	±0.87%	16.83%	18.41%	5.03%	3.65%	-	-	-	13.29%	5.28%	1.83%	1.56%
**7989**	0.11%	15.02%	11.74%	15.49%	14.08%	±1.67%	16.60%	15.11%	16.35%	16.02%	±0.65%	15.48%	11.89%	5.02%	4.09%	-	-	-	14.97%	5.26%	2.02%	0.61%
**8079**	0.00%	90.31%	90.98%	90.77%	90.69%	±0.28%	89.08%	91.70%	91.71%	90.83%	±1.23%	90.76%	93.33%	56.79%	57.39%	-	-	-	93.30%	69.90%	38.25%	33.33%
**8110**	0.88%	93.98%	94.42%	92.71%	93.70%	±0.73%	93.32%	93.07%	94.08%	93.49%	±0.43%	92.71%	91.43%	57.88%	59.86%	-	-	-	93.34%	77.05%	49.17%	45.94%
**8237**	1.14%	12.47%	9.24%	11.63%	11.11%	±1.37%	11.01%	14.24%	12.83%	12.70%	±1.32%	11.58%	12.65%	0.00%	0.00%	0.00%	-	-	14.23%	3.33%	0.62%	1.70%
**8440**	0.13%	11.60%	11.61%	11.97%	11.73%	±0.17%	10.33%	10.77%	11.33%	10.81%	±0.41%	11.97%	0.00%	0.14%	0.05%	0.00%	0.00%	0.00%	12.79%	3.79%	1.65%	1.41%
**8564**	0.17%	20.75%	20.46%	21.84%	21.02%	±0.60%	18.78%	21.30%	20.69%	20.26%	±1.07%	21.84%	26.30%	0.47%	0.37%	0.00%	0.00%	0.00%	22.01%	8.66%	3.90%	3.32%
**8571**	0.08%	78.78%	81.29%	79.92%	80.00%	±1.02%	74.37%	80.79%	79.78%	78.31%	±2.82%	79.92%	71.35%	3.11%	3.64%	0.00%	8.25%	3.14%	83.81%	52.63%	29.10%	24.93%
**8621**	0.33%	81.29%	80.77%	81.20%	81.09%	±0.23%	78.20%	81.56%	81.09%	80.28%	±1.49%	81.20%	74.67%	13.13%	5.77%	14.67%	6.81%	0.00%	83.78%	45.15%	23.94%	21.66%
**8710**	0.93%	46.92%	47.72%	48.56%	47.73%	±0.67%	44.28%	45.76%	47.55%	45.87%	±1.34%	48.56%	43.51%	8.72%	7.81%	2.19%	9.60%	0.00%	51.12%	20.02%	9.18%	7.53%
**8947**	0.73%	48.75%	48.47%	48.51%	48.58%	±0.12%	46.15%	48.18%	47.87%	47.40%	±0.90%	48.53%	46.96%	21.69%	16.88%	14.27%	31.82%	15.85%	50.72%	20.74%	10.28%	8.66%
**8975**	0.63%	89.56%	92.55%	92.24%	91.45%	±1.34%	88.28%	92.64%	91.55%	90.82%	±1.85%	92.24%	85.81%	69.14%	71.68%	85.57%	88.95%	77.95%	90.96%	70.39%	42.76%	39.47%
**8989**	0.53%	69.12%	71.52%	71.11%	70.58%	±1.05%	67.93%	72.36%	71.64%	70.65%	±1.94%	71.10%	64.09%	37.38%	34.47%	51.20%	41.14%	38.28%	73.50%	30.31%	14.60%	12.55%
**8034**	1.24%	44.03%	45.19%	46.40%	45.21%	±0.97%	43.87%	43.34%	46.44%	44.55%	±1.35%	46.38%	54.65%	16.50%	15.71%	-	-	-	44.10%	16.08%	7.37%	6.99%

Supplementary Table S3. Significant m5C modification sites in the HIV-1 sense epitranscriptome.

		Jurkat cells with HIV-GFPΔEnv/VSV-G						Jurkat cells with HIV-GFPΔEnv/VSV-G and cART					(ribosome depletion method) 11B/H		CD4 T-cells with NL4-3 HIV	CD4 supernatant with NL4-3 HIV	Patient CD4 T-cells			m6A Chemical Knockdown			
Base	T7-InVitro	7C minus T7	11E minus T7	11B/H minus T7	HIV average	HIV stdev	m6A Adjacent ?	7E minus T7	11F minus T7	11C/I minus T7	HIV+cART average	HIV+cART stdev	Poly-A-selection minus T7	RNaseH reaction minus T7	10D minus T7	14H minus T7	P5S2 minus T7	P6S minus T7	P8 minus T7	STM2457 0 μM minus T7	STM2457 7.5 μM minus T7	STM2457 30 μM minus T7	STM2457 60 μM minus T7
**1551**	20.54%	0.00%	23.90%	9.09%	11.00%	±9.85%	No	0.00%	12.79%	5.77%	6.19%	±5.23%	0.00%	19.46%	12.79%	0.00%	-	-	-	3.60%	0.00%	11.72%	15.46%
**8114**	12.58%	16.75%	28.99%	29.58%	25.11%	±5.91%	No	19.12%	24.03%	26.92%	23.35%	±3.22%	29.70%	20.75%	17.31%	16.35%	-	-	-	17.47%	29.94%	5.46%	11.89%
**8976**	3.40%	3.69%	14.81%	16.00%	11.50%	±5.54%	Yes	4.37%	10.59%	14.40%	9.79%	±4.13%	16.02%	9.10%	10.06%	9.28%	11.50%	3.58%	3.53%	12.44%	10.69%	3.28%	3.92%
**8990**	7.31%	24.30%	33.69%	34.77%	30.92%	±4.70%	Yes	25.22%	30.32%	33.84%	29.79%	±3.54%	34.78%	11.92%	16.93%	14.41%	35.00%	16.42%	9.56%	33.93%	14.18%	4.34%	4.06%

Supplementary Table S4. Significant inosine modification sites in the HIV-1 sense epitranscriptome.

		Jurkat cells with HIV-GFPΔEnv/VSV-G					Jurkat cells with HIV-GFPΔEnv/VSV-G and cART					Jurkats cells (ribosome depletion method)		CD4 T-cells with NL4-3 HIV	CD4 supernatant with NL4-3 HIV	Patient CD4 T-cells			m6A Chemical Knockdown			
Base	T7-InVitro	7C minus T7	11E minus T7	11B/H minus T7	HIV average 7C,11E,11B/H	HIV stdev	7E minus T7	11F minus T7	11C/I minus T7	HIV+cART average	HIV+cART stdev	Poly-A-selection minus T7	RNaseH reaction minus T7	10D minus T7	14H minus T7	P5S2 minus T7	P6S minus T7	P8 minus T7	STM2457 0 μM minus T7	STM2457 7.5 μM minus T7	STM2457 30 μM minus T7	STM2457 60 μM minus T7
**8037**	2.14%	9.89%	14.93%	13.35%	12.72%	±2.11%	10.29%	14.09%	11.05%	12.19%	±1.90%	13.36%	10.36%	3.55%	4.92%	-	-	-	10.81%	5.08%	1.84%	2.51%
**8568**	0.56%	5.08%	12.83%	20.09%	12.67%	±6.13%	5.60%	12.30%	18.36%	8.95%	±3.35%	20.13%	19.44%	0.12%	0.20%	0.00%	7.77%	1.13%	15.94%	9.30%	3.36%	2.97%

Supplementary Table S5. Significant 2'-O-methylation modification sites in the HIV-1 sense epitranscriptome.

		Jurkat cells with HIV-GFPΔEnv/VSV-G					Jurkat cells with HIV-GFPΔEnv/VSV-G and cART					Jurkats cells (ribosome depletion method)		CD4 T-cells with NL4-3 HIV	CD4 supernatant with NL4-3 HIV	Patient CD4 T-cells			m6A Chemical Knockdown			
Base		7C	11E	11B/H	HIV average	HIV stdev	7E	11F	11C/I	HIV+cART average	HIV+cART stdev	Poly-A-selection	RNaseH reaction	10D minus T7	11H minus T7	P5S2 minus T7	P6S minus T7	P8 minus T7	STM2457 0 μM minus T7	STM2457 7.5 μM minus T7	STM2457 30 μM minus T7	STM2457 60 μM minus T7
**1749**	6.82%	0.00%	9.85%	25.00%	11.62%	±10.28%	0.00%	0.00%	7.47%	2.49%	±3.52%	23.18%	26.52%	5.68%	0.00%	-	-	-	9.46%	0.00%	0.00%	0.00%
**3107**	4.72%	21.18%	-	1.53%	11.36%	±9.82%	16.15%	0.00%	0.00%	5.38%	±7.61%	0.00%	0.00%	0.00%	0.00%	-	-	-	0.00%	0.28%	0.00%	0.00%
**9162**	7.41%	12.96%	18.81%	28.46%	20.08%	±6.39%	18.32%	23.16%	25.52%	22.33%	±3.00%	28.47%	21.53%	20.02%	26.28%	41.42%	31.16%	26.83%	23.57%	30.23%	24.46%	29.58%
**9164**	1.27%	31.69%	6.97%	7.00%	15.22%	±11.65%	26.43%	13.06%	5.20%	14.90%	±8.76%	7.00%	11.32%	4.83%	6.08%	0.29%	6.96%	12.08%	6.60%	3.09%	6.56%	2.43%

Supplementary Table S6. Significant pseudouridine modification sites in HIV-1 sense epitranscriptome (low threshold).

		Jurkat cells with HIV-GFPΔEnv/VSV-G					Jurkat cells with HIV-GFPΔEnv/VSV-G and cART					Jurkats cells (ribosome depletion method)		CD4 T-cells with NL4-3 HIV	supernatant with NL4-3 HIV	Patient CD4 T-cells			m6A Chemical Knockdown			
Base	T7-InVitro	7C minus T7	11E minus T7	11B/H minus T7	HIV average 7C,11B,11E,11H	HIV stdev	7E minus T7	11F minus T7	11C/I minus T7	HIV+cART average 7E,11C,11F,11I	HIV+cART stdev	Poly-A-selection minus T7	RNaseH reaction minus T7	10D minus T7	14H minus T7	P5S2 minus T7	P6S minus T7	P8 minus T7	STM2457 0 μM minus T7	STM2457 7.5 μM minus T7	STM2457 30 μM minus T7	STM2457 60 μM minus T7
**637**	3.78%	1.51%	4.22%	12.22%	5.98%	±4.54%	0.00%	0.00%	0.00%	0.00%	±0.00%	0.00%	15.26%	0.00%	0.00%	-	-	-	4.86%	0.00%	0.00%	0.27%
**1214**	41.41%	6.82%	0.00%	10.97%	5.93%	±4.52%	0.00%	0.00%	0.00%	0.00%	±0.00%	-	5.25%	0.00%	1.26%	-	-	-	0.00%	0.00%	0.00%	3.03%
**1422**	1.39%	0.00%	18.61%	3.37%	7.32%	±8.09%	0.00%	0.00%	0.00%	0.00%	±0.00%	0.00%	7.70%	0.00%	0.00%	-	-	-	0.00%	0.00%	0.53%	3.37%
**1646**	4.91%	0.00%	22.36%	0.00%	7.45%	±10.54%	0.00%	0.00%	0.00%	0.00%	±0.00%	0.00%	0.00%	0.97%	0.00%	-	-	-	2.93%	0.00%	2.64%	0.00%
**2724**	10.23%	0.00%	3.57%	12.50%	5.36%	±5.26%	0.00%	0.00%	0.00%	0.00%	±0.00%	0.00%	0.00%	0.00%	1.41%	-	-	-	4.92%	11.99%	0.00%	0.00%
**3780**	7.45%	5.29%	10.20%	0.00%	5.16%	±4.16%	0.00%	2.55%	0.00%	0.85%	±1.20%	-	0.00%	0.88%	0.00%	-	-	-	0.00%	0.55%	0.00%	8.18%
**4451**	0.81%	0.00%	19.19%	0.00%	6.40%	±9.04%	0.00%	0.00%	0.00%	0.00%	±0.00%	0.00%	0.00%	0.00%	0.57%	-	-	-	0.00%	0.00%	0.00%	10.30%

Supplementary Table S7. Significant differential modifications in transcript splice isoforms.

Base #	Mod	gag / pol	vif	vpr	tat	rev	vpu / env	nef	Diff with gag/pol	vif diff	vpr diff	tat diff	rev diff	vpu diff	nef diff
**265**	m^5C	21.76%	3.24%	21.34%	15.91%	18.21%	13.53%	17.58%	18.52%	-18.52%	-0.42%	-5.85%	-3.54%	-8.23%	-4.18%
**5006**	m^6A	10.20%	13.44%	14.43%	30.29%	2.46%	5.41%	11.19%	20.09%	3.24%	4.23%	20.09%	-7.74%	-4.79%	0.99%
**5160**	m^6A	4.84%	20.42%	26.67%	-	-	-	-	21.84%	15.58%	21.84%	-	-	-	-
**5434**	m^6A	1.77%	2.38%	3.31%	12.22%	-	-	-	10.45%	0.61%	1.54%	10.45%	-	-	-
**5750**	2'-O-Met	2.25%	9.52%	12.33%	-	-	3.67%	-	10.08%	7.27%	10.08%	-	-	1.41%	-
**6242**	Psi	1.92%	6.77%	12.56%	-	-	4.21%	-	10.64%	4.85%	10.64%	-	-	2.30%	-
**8851**	Psi	0.26%	11.47%	3.00%	5.94%	10.56%	5.12%	7.00%	11.21%	11.21%	2.75%	5.68%	10.31%	4.87%	6.75%
**8975**	m^6A	94.16%	89.64%	92.79%	87.02%	82.33%	90.38%	84.82%	11.83%	-4.51%	-1.37%	-7.13%	-11.83%	-3.78%	-9.33%
**9008**	m^5C	6.12%	8.28%	17.54%	4.04%	4.65%	5.30%	4.35%	11.42%	2.16%	11.42%	-2.08%	-1.47%	-0.82%	-1.78%
**9019**	m^5C	1.49%	5.87%	12.15%	3.60%	7.28%	1.85%	3.00%	10.65%	4.37%	10.65%	2.11%	5.79%	0.36%	1.51%
**9166**	2'-O-Met	10.08%	4.88%	20.21%	7.66%	5.87%	7.67%	9.16%	10.13%	-5.20%	10.13%	-2.42%	-4.21%	-2.41%	-0.92%

Supplementary Table S8. Modifications that fall within a known splicing regulatory element.

Base #	Mod	gag / pol	vif	vpr	tat	rev	vpu / env	nef	Notes
265	m^5C	21.76%	3.24%	21.34%	15.91%	18.21%	13.53%	17.58%	Within ESE U5, involved in promoting splicing at D1 (end of 5' LTR)
5006	m^6A	10.20%	13.44%	14.43%	30.29%	2.46%	5.41%	11.19%	Within HIVE3D3, involved in silencing splicing at D3 (3' end of S2 splice donor)
7983	m^6A	13.40%	20.41%	12.94%	16.90%	18.18%	16.80%	17.90%	Within ESE-3, involved in promoting splicing at A7 (3' region used by *tat*/*rev*/*nef* isoforms)
7989	m^6A	14.46%	14.47%	18.60%	16.90%	16.70%	16.45%	17.93%	

Supplementary Table S9. Significant modification sites in the NL4-3-GFP HIV-1 antisense epitranscriptome.

Whole genome, mod% > 20%, at least 10x coverage

		m6A				m5C				Pseudouridine				Inosine				Inosine		
	**Base**	**Jurkats**	**T7**	**Diff**	**J count**	**Jurkats**	**T7**	**Diff**	**J count**	**Jurkats**	**T7**	**Diff**	**J count**	**Jurkats**	**T7**	**Diff**	**J count**	**Jurkats**	**T7**	**Diff**
A	**95**	0.00%	0.00%	0.00%	0	0.00%	0.00%	0.00%	0	0.00%	0.00%	0.00%	0	30.00%	1.59%	28.41%	10	0.00%	0.00%	0.00%
A	**118**	0.00%	0.00%	0.00%	0	0.00%	0.00%	0.00%	0	0.00%	0.00%	0.00%	0	30.00%	0.00%	30.00%	10	0.00%	0.00%	0.00%
T	**706**	0.00%	0.00%	0.00%	0	0.00%	0.00%	0.00%	0	0.00%	0.00%	0.00%	10	0.00%	0.00%	0.00%	0	20.00%	0.00%	20.00%
T	**728**	0.00%	0.00%	0.00%	0	0.00%	0.00%	0.00%	0	0.00%	1.82%	0.00%	10	0.00%	0.00%	0.00%	0	20.00%	0.00%	20.00%
A	**1056**	0.00%	0.00%	0.00%	0	0.00%	0.00%	0.00%	0	0.00%	0.00%	0.00%	0	25.00%	4.65%	20.35%	12	0.00%	0.00%	0.00%
T	**1070**	0.00%	0.00%	0.00%	0	0.00%	0.00%	0.00%	0	0.00%	0.00%	0.00%	10	0.00%	0.00%	0.00%	0	20.00%	0.00%	20.00%
T	**1093**	0.00%	0.00%	0.00%	0	0.00%	0.00%	0.00%	0	53.85%	0.00%	53.85%	13	0.00%	0.00%	0.00%	0	0.00%	0.00%	0.00%
T	**1564**	0.00%	0.00%	0.00%	0	0.00%	0.00%	0.00%	0	0.00%	0.00%	0.00%	12	0.00%	0.00%	0.00%	0	25.00%	3.85%	21.15%
C	**1717**	0.00%	0.00%	0.00%	0	30.00%	0.00%	30.00%	10	0.00%	0.00%	0.00%	0	0.00%	0.00%	0.00%	0	0.00%	0.00%	0.00%
A	**2339**	0.00%	0.00%	0.00%	0	0.00%	0.00%	0.00%	0	0.00%	0.00%	0.00%	0	26.67%	2.33%	24.34%	15	0.00%	0.00%	0.00%
C	**2361**	0.00%	0.00%	0.00%	0	0.00%	0.17%	0.00%	13	0.00%	0.00%	0.00%	0	0.00%	0.00%	0.00%	0	30.77%	5.36%	25.41%
C	**2903**	0.00%	0.00%	0.00%	0	0.00%	0.00%	0.00%	12	0.00%	0.00%	0.00%	0	0.00%	0.00%	0.00%	0	33.33%	7.39%	25.94%
T	**3104**	0.00%	0.00%	0.00%	0	0.00%	0.00%	0.00%	0	21.43%	0.63%	20.80%	14	0.00%	0.00%	0.00%	0	0.00%	0.00%	0.00%
T	**3145**	0.00%	0.00%	0.00%	0	0.00%	0.00%	0.00%	0	0.00%	0.61%	0.00%	13	0.00%	0.00%	0.00%	0	23.08%	1.23%	21.85%
T	**3153**	0.00%	0.00%	0.00%	0	0.00%	0.00%	0.00%	0	0.00%	0.00%	0.00%	13	0.00%	0.00%	0.00%	0	23.08%	2.99%	20.08%
A	**3311**	0.00%	0.00%	0.00%	0	0.00%	0.00%	0.00%	0	0.00%	0.00%	0.00%	0	23.08%	1.20%	21.87%	13	0.00%	0.00%	0.00%
T	**3504**	0.00%	0.00%	0.00%	0	0.00%	0.00%	0.00%	0	0.00%	3.56%	0.00%	12	0.00%	0.00%	0.00%	0	33.33%	1.78%	31.56%
T	**4179**	0.00%	0.00%	0.00%	0	0.00%	0.00%	0.00%	0	0.00%	0.00%	0.00%	10	0.00%	0.00%	0.00%	0	30.00%	2.99%	27.01%
T	**4687**	0.00%	0.00%	0.00%	0	0.00%	0.00%	0.00%	0	0.00%	0.00%	0.00%	13	0.00%	0.00%	0.00%	0	38.46%	4.76%	33.70%
A	**6667**	0.00%	0.00%	0.00%	0	0.00%	0.00%	0.00%	0	0.00%	0.00%	0.00%	0	24.14%	1.38%	22.76%	29	0.00%	0.46%	0.00%
C	**8021**	0.00%	0.00%	0.00%	0	23.81%	3.81%	20.00%	21	0.00%	0.00%	0.00%	0	0.00%	0.00%	0.00%	0	0.00%	0.95%	0.00%
C	**8620**	0.00%	0.00%	0.00%	0	7.69%	0.74%	6.95%	13	0.00%	0.00%	0.00%	0	0.00%	0.00%	0.00%	0	23.08%	2.58%	20.49%
C	**8625**	0.00%	0.00%	0.00%	0	0.00%	0.70%	0.00%	20	0.00%	0.00%	0.00%	0	0.00%	0.00%	0.00%	0	25.00%	4.58%	20.42%
A	**8804**	0.00%	0.00%	0.00%	0	0.00%	0.00%	0.00%	0	0.00%	0.00%	0.00%	0	22.73%	2.54%	20.18%	22	4.55%	0.00%	4.55%
A	**9051**	0.00%	0.00%	0.00%	0	0.00%	0.00%	0.00%	0	0.00%	0.00%	0.00%	0	23.08%	2.37%	20.71%	13	0.00%	0.00%	0.00%
C	**9071**	0.00%	0.00%	0.00%	0	27.27%	2.83%	24.44%	11	0.00%	0.00%	0.00%	0	0.00%	0.00%	0.00%	0	0.00%	0.81%	0.00%
A	**9151**	0.00%	0.00%	0.00%	0	0.00%	0.00%	0.00%	0	0.00%	0.00%	0.00%	0	25.00%	0.92%	24.08%	12	0.00%	0.00%	0.00%

ASP gene only, mod% > 10%

		m6A				m5C				Pseudouridine				Inosine				Inosine		
	**Base**	**Jurkats**	**T7**	**Diff**	**J count**	**Jurkats**	**T7**	**Diff**	**J count**	**Jurkats**	**T7**	**Diff**	**J count**	**Jurkats**	**T7**	**Diff**	**J count**	**Jurkats**	**T7**	**Diff**
A	**6911**	0.00%	0.00%	0.00%	0	0.00%	0.00%	0.00%	0	0.00%	0.00%	0.00%	0	23.33%	0.30%	23.04%	30	0.00%	0.00%	0.00%
A	**6916**	0.00%	0.00%	0.00%	0	0.00%	0.00%	0.00%	0	0.00%	0.00%	0.00%	0	10.00%	0.00%	10.00%	30	0.00%	0.27%	0.00%
A	**6949**	0.00%	0.00%	0.00%	0	0.00%	0.00%	0.00%	0	0.00%	0.00%	0.00%	0	10.00%	0.00%	10.00%	40	0.00%	0.00%	0.00%
A	**7020**	0.00%	0.00%	0.00%	0	0.00%	0.00%	0.00%	0	0.00%	0.00%	0.00%	0	15.15%	3.42%	11.73%	33	0.00%	0.00%	0.00%
A	**7032**	0.00%	0.00%	0.00%	0	0.00%	0.00%	0.00%	0	0.00%	0.00%	0.00%	0	19.35%	3.27%	16.08%	31	3.23%	0.47%	2.76%
A	**7122**	0.00%	0.00%	0.00%	0	0.00%	0.00%	0.00%	0	0.00%	0.00%	0.00%	0	11.36%	0.39%	10.97%	44	0.00%	0.00%	0.00%
A	**7163**	0.00%	0.00%	0.00%	0	0.00%	0.00%	0.00%	0	0.00%	0.00%	0.00%	0	10.26%	0.00%	10.26%	39	0.00%	0.00%	0.00%
T	**7177**	0.00%	0.00%	0.00%	0	0.00%	0.00%	0.00%	0	0.00%	0.66%	0.00%	26	0.00%	0.00%	0.00%	0	23.08%	12.58%	10.49%
G	**7180**	0.00%	0.00%	0.00%	0	0.00%	0.00%	0.00%	0	0.00%	0.00%	0.00%	0	0.00%	0.00%	0.00%	0	16.67%	5.00%	11.67%
T	**7182**	0.00%	0.00%	0.00%	0	0.00%	0.00%	0.00%	0	0.00%	0.00%	0.00%	30	0.00%	0.00%	0.00%	0	10.00%	0.00%	10.00%
A	**7312**	0.00%	0.00%	0.00%	0	0.00%	0.00%	0.00%	0	0.00%	0.00%	0.00%	0	12.12%	0.00%	12.12%	33	0.00%	0.00%	0.00%
T	**7342**	0.00%	0.00%	0.00%	0	0.00%	0.00%	0.00%	0	0.00%	0.00%	0.00%	29	0.00%	0.00%	0.00%	0	20.69%	7.25%	13.44%
A	**7470**	17.50%	0.00%	17.50%	40	0.00%	0.00%	0.00%	0	0.00%	0.00%	0.00%	0	0.00%	0.00%	0.00%	40	0.00%	0.80%	0.00%

Supplementary Table S10. Comparison of modification frequencies between new (v5.2.0) and old (v5.1.0) Oxford Nanopore Technologies modification-calling algorithms.

Base	Mod Type	Algorithm	7C	11E	11B/H	HIV average	T7-InVitro	7C minus T7	11E minus T7	11B/H minus T7	HIV average
448	ino	v5.1.0	3.10%	0.00%	36.36%	13.16%	6.13%	0.00%	0.00%	30.23%	10.08%
		v5.2.0	1.58%	0.00%	0.00%	0.53%	1.27%	0.31%	0.00%	0.00%	0.10%
495	ino	v5.1.0	2.40%	16.67%	20.00%	13.02%	2.92%	0.00%	13.74%	17.08%	10.27%
		v5.2.0	0.69%	0.00%	0.00%	0.23%	1.66%	0.00%	0.00%	0.00%	0.00%
1,483	psi	v5.1.0	15.92%	53.33%	51.85%	40.37%	27.68%	0.00%	25.65%	24.17%	16.61%
		v5.2.0	5.01%	10.00%	9.09%	8.04%	4.21%	0.80%	5.79%	4.88%	3.82%
2,547	m^5C	v5.1.0	16.34%	33.33%	14.29%	21.32%	8.08%	8.26%	25.26%	6.21%	13.24%
		v5.2.0	6.03%	13.33%	8.33%	9.23%	10.64%	0.00%	2.69%	0.00%	0.90%
2,607	psi	v5.1.0	28.83%	76.67%	75.00%	60.17%	60.52%	0.00%	16.15%	14.48%	10.21%
		v5.2.0	1.30%	5.13%	12.00%	6.14%	3.02%	0.00%	2.11%	8.98%	3.70%
3,088	ino	v5.1.0	2.09%	22.22%	23.81%	16.04%	5.32%	0.00%	16.90%	18.49%	11.80%
		v5.2.0	0.85%	0.00%	13.33%	4.73%	1.43%	0.00%	0.00%	11.90%	3.97%
3,647	ino	v5.1.0	0.63%	17.78%	23.26%	13.89%	3.90%	0.00%	13.88%	19.36%	11.08%
		v5.2.0	0.22%	0.00%	0.00%	0.07%	0.94%	0.00%	0.00%	0.00%	0.00%
4,305	m^5C	v5.1.0	13.80%	17.65%	4.44%	11.97%	0.84%	12.96%	16.81%	3.60%	11.12%
		v5.2.0	3.08%	0.00%	2.13%	1.73%	1.37%	1.71%	0.00%	0.76%	1.23%
5,253	psi	v5.1.0	15.95%	34.04%	27.97%	25.99%	14.71%	1.24%	19.33%	13.26%	11.28%
		v5.2.0	3.66%	12.50%	9.14%	8.43%	4.35%	0.00%	8.15%	4.80%	4.32%
7,501	m^5C	v5.1.0	71.72%	12.50%	14.83%	33.02%	32.77%	38.95%	0.00%	0.00%	12.98%
		v5.2.0	42.63%	8.47%	7.62%	19.57%	17.92%	24.71%	0.00%	0.00%	8.24%
7,966	ino	v5.1.0	7.97%	37.60%	37.12%	27.57%	21.60%	0.00%	16.00%	15.52%	10.50%
		v5.2.0	9.40%	1.27%	1.70%	4.13%	2.73%	6.67%	0.00%	0.00%	2.22%
8,322	psi	v5.1.0	10.13%	9.22%	18.71%	12.69%	1.85%	8.28%	7.37%	16.86%	10.84%
		v5.2.0	2.78%	0.00%	2.89%	1.89%	0.41%	2.37%	0.00%	2.48%	1.62%
8,514	m^5C	v5.1.0	69.24%	71.35%	67.78%	69.46%	54.31%	14.93%	17.05%	13.48%	15.15%
		v5.2.0	15.54%	27.55%	18.52%	20.54%	22.11%	0.00%	5.44%	0.00%	1.81%
9,171	psi	v5.1.0	25.97%	4.66%	8.51%	13.05%	0.82%	25.15%	3.84%	7.69%	12.23%
		v5.2.0	1.75%	1.39%	1.13%	1.42%	0.17%	1.58%	1.23%	0.96%	1.26%

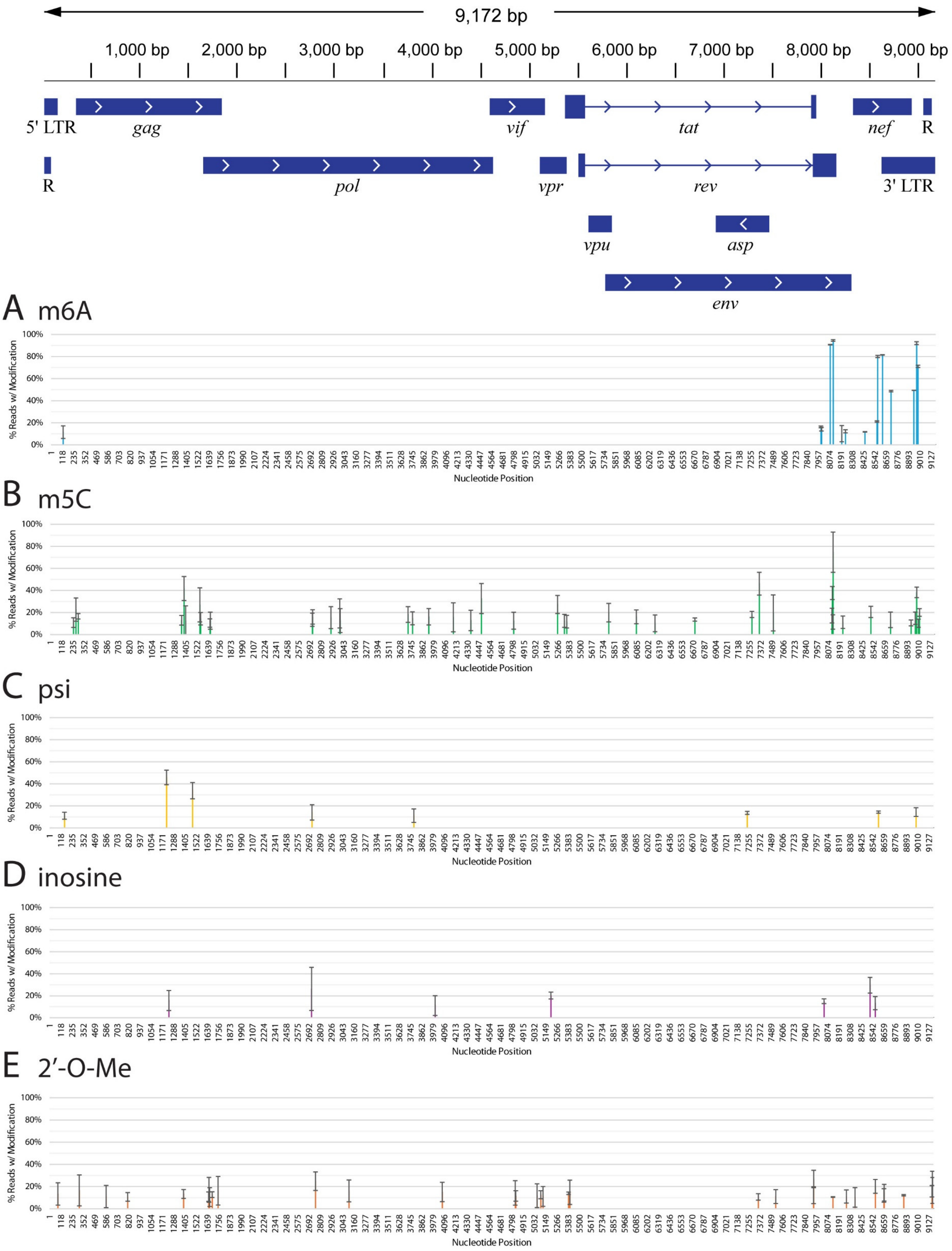

Supplementary Fig. S1. **Uncorrected nanopore modification-calling results for HIV-1 viral RNA from Jurkat cells**. X-axis represents individual nucleotides in the HIV-1 genome. Y-axis represents percentage of reads that contained the mutation of interest for that nucleotide. (**A**) m^6A (blue), (**B**) m^5C (green), (**C**) pseudouridine (psi) (yellow), (**D**) inosine (purple), and (**E**) 2'-*O*-methylation (orange). Results are the average of three separate biological replicates. Error bars are standard deviation. Only results with percentages above 10% are shown.

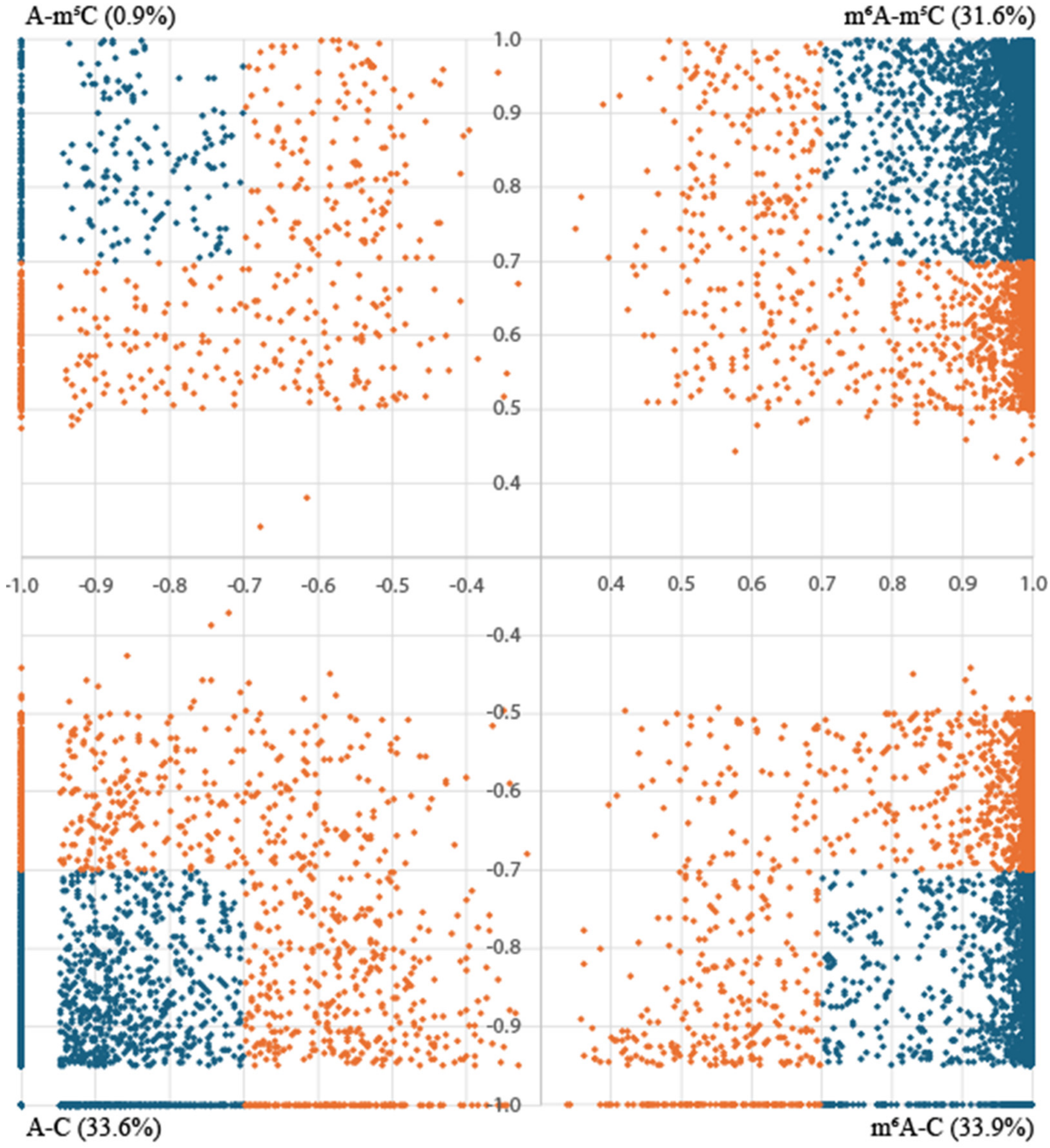

<u>Supplementary Fig. S2</u>. Adjacent m^{6}A-m^{5}C Distribution. Distribution of reads at positions 8989 (A) and 8990 (C) in sample 7C. For each point, the X-axis shows the probability of 8989-A/m^{6}A modification call accuracy. Reads called as A have had the sign of the probability reversed. The Y-axis shows the probability of the 8990-C/m^{5}C modification call accuracy Reads called as C have had the sign of the probability flipped. Points marked in blue are those in which the read has at least a 70% probability of accuracy for both positions. Points marked in orange fall outside this cutoff criteria and are not considered.

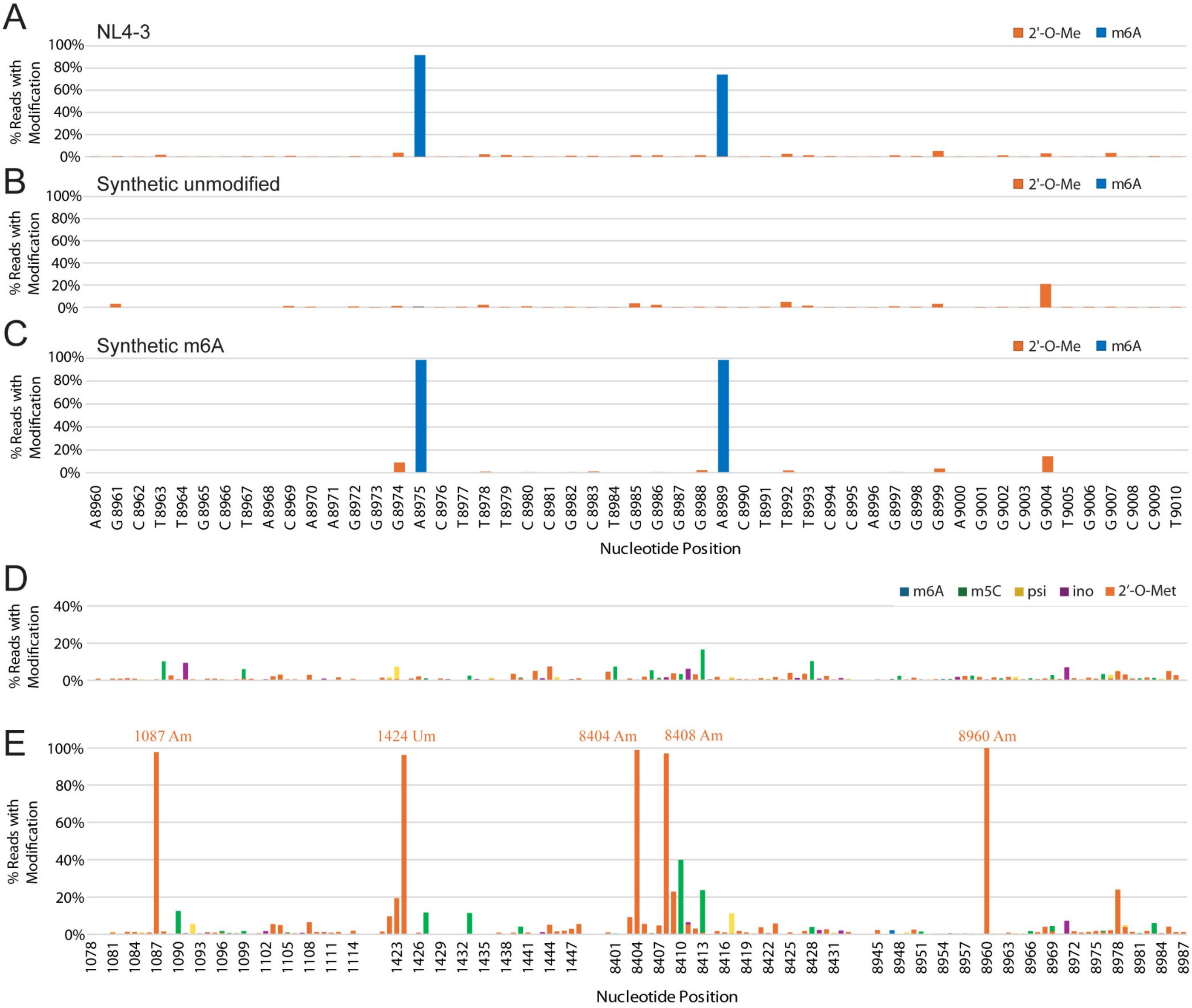

<u>**Supplementary Fig. S3**</u>**. 2'-*O*-methylation calling in HIV-1 RNA and synthetic RNA fragments.** Comparison of modification calling between NL4-3 from Jurkat cells (**A**) and two synthetic HIV-1 RNA fragments, one unmodified (**B**) and one bearing m^6A (**C**) at two DRACH motifs. The nucleotide position corresponding to the NL4-3 genome is indicated on the x-axis. All modifications called in panel B are considered incorrect while modifications called in the lower panel, besides m^6A at position 8975 and 8989, are considered incorrect. Comparison of modification calling between *in vitro* transcribed RNA containing no modified bases (**D**) and synthetic HIV-1 RNA fragments (**E**). Four ~60nt RNA oligonucleotides containing 2'-*O*-methylated based at reported positions were synthesized, mixed, and sequenced. All modifications called in panel D are considered incorrect while modifications called in the lower panel, besides those that are labeled, are considered incorrect.

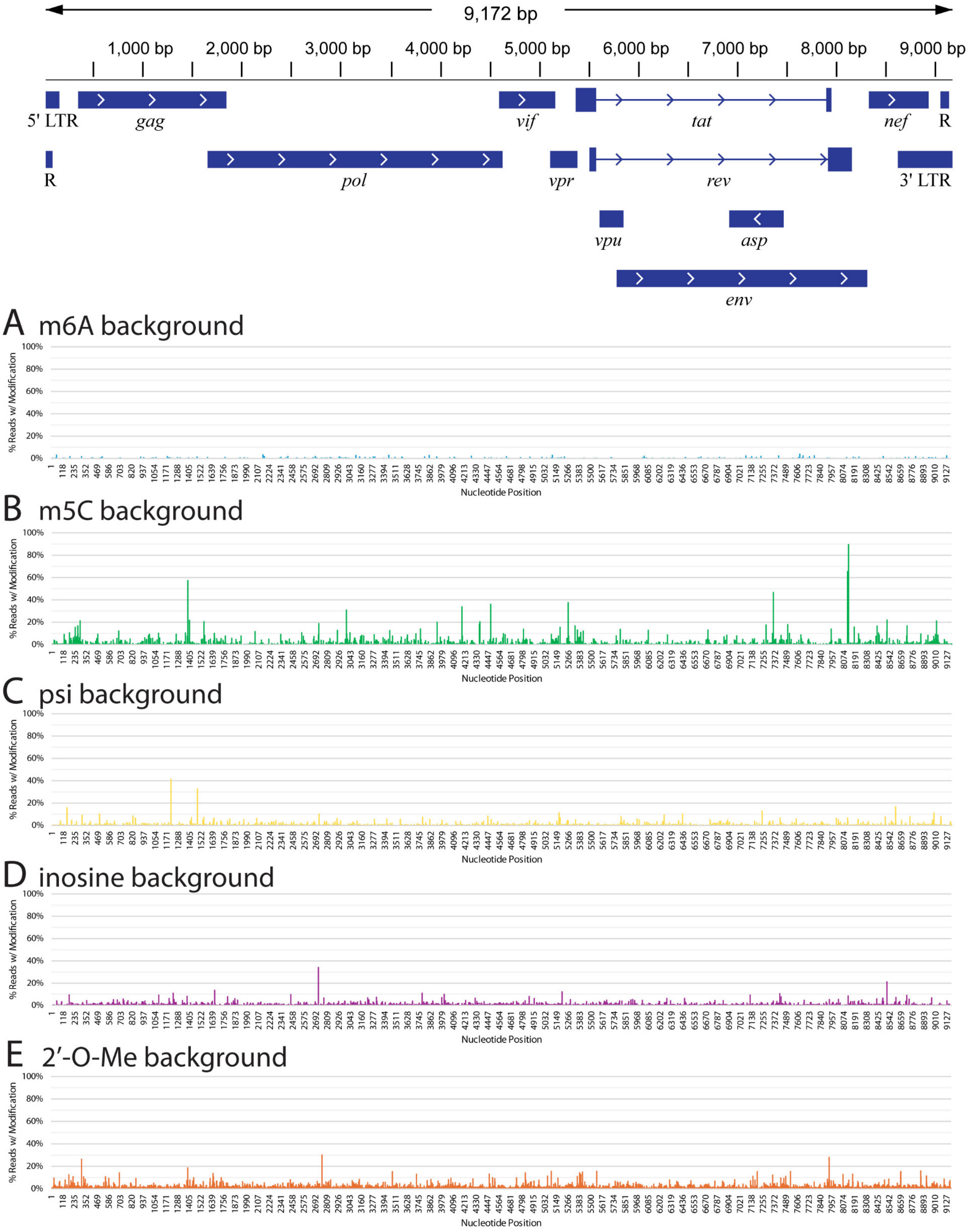

<u>Supplementary Fig. S4</u>. Modification calling from nanopore sequencing of *in vitro* transcribed NL4-3 HIV-1 RNA fragments used for baseline correction. X-axis represents individual nucleotides in the HIV-1 genome. Y-axis represents percentage of reads that contained the mutation of interest for that nucleotide. m^6A (blue), m^5C (green), pseudouridine (psi) (yellow), inosine (purple) and 2'-*O*-methylation (orange).

A

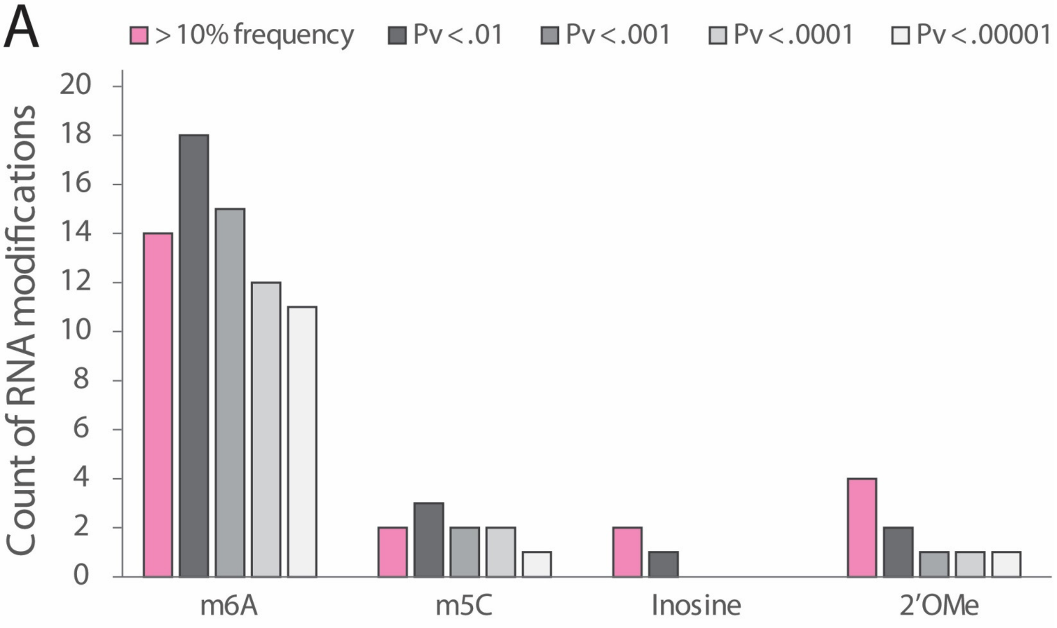

B

NT	mod	7C corrected	11E corrected	11BH corrected	7C P value	11E P value	11BH P value
125	m6A	3.09%	13.66%	16.37%	1.0E+00	1.8E-03	1.2E-04
7983	m6A	13.42%	15.65%	16.63%	1.8E-03	2.0E-04	9.9E-05
7989	m6A	14.93%	11.63%	16.46%	2.7E-04	7.9E-03	3.6E-05
8079	m6A	89.60%	90.26%	90.49%	2.9E-44	2.5E-45	2.5E-45
8110	m6A	94.03%	94.62%	93.12%	3.9E-50	3.9E-50	7.7E-49
8237	m6A	12.21%	8.86%	10.96%	4.0E-03	2.5E-01	2.4E-02
8440	m6A	10.01%	11.22%	11.74%	2.4E-02	9.4E-03	4.0E-03
8564	m6A	20.68%	20.42%	22.05%	8.0E-07	8.0E-07	3.2E-07
8571	m6A	78.58%	81.16%	78.07%	1.4E-36	3.7E-38	8.1E-36
8621	m6A	80.56%	80.17%	76.50%	6.6E-37	4.2E-36	8.4E-34
8710	m6A	46.78%	47.76%	46.90%	1.3E-16	4.6E-17	1.3E-16
8947	m6A	48.64%	48.54%	46.85%	1.6E-17	4.6E-17	1.3E-16
8975	m6A	88.85%	91.99%	91.49%	2.9E-44	1.3E-47	1.3E-47
8989	m6A	68.26%	70.81%	72.48%	8.9E-27	4.8E-28	2.3E-29
1551	m5C	-13.30%	23.98%	8.11%	6.3E-02	4.7E-02	5.6E-01
8114	m5C	16.55%	29.03%	30.58%	1.0E-02	1.5E-05	7.4E-06
8037	Ino	9.80%	14.72%	12.99%	4.1E-02	1.3E-03	2.4E-03
8568	Ino	10.83%	10.82%	21.04%	1.6E-02	1.6E-02	2.5E-04
1749	2OMe	-6.85%	9.47%	24.62%	1.0E+00	4.7E-01	1.5E-02
3107	2OMe	21.18%	-	1.53%	2.5E-04	-	1.0E+00
9162	2OMe	12.71%	16.96%	29.45%	4.9E-01	5.4E-03	1.1E-05
9164	2OMe	31.69%	6.97%	1.92%	2.1E-10	1.0E+00	1.0E+00

Supplementary Fig. S5. Modkit dmr P-value analysis. P-values generated by Nanopore's modkit program in dmr mode to estimate the difference in modification between a control sample (*in vitro* transcribed RNA containing no modifications) and an experimental sample (extracted from infected cells). **(A)** The number of modifications called on HIV-1 transcripts for each P-value cutoff (0.01, 0.001, 0.0001, 0.00001) as well as the 10% frequency cutoff used in the manuscript. **(B)** Comparison of sample modification frequency and P-values at specific nucleotides within the HIV-1 transcriptome. Sample modification frequency varies slightly from those reported elsewhere in the manuscript due to modkit utilizing a subsampling algorithm as part of the dmr analysis.

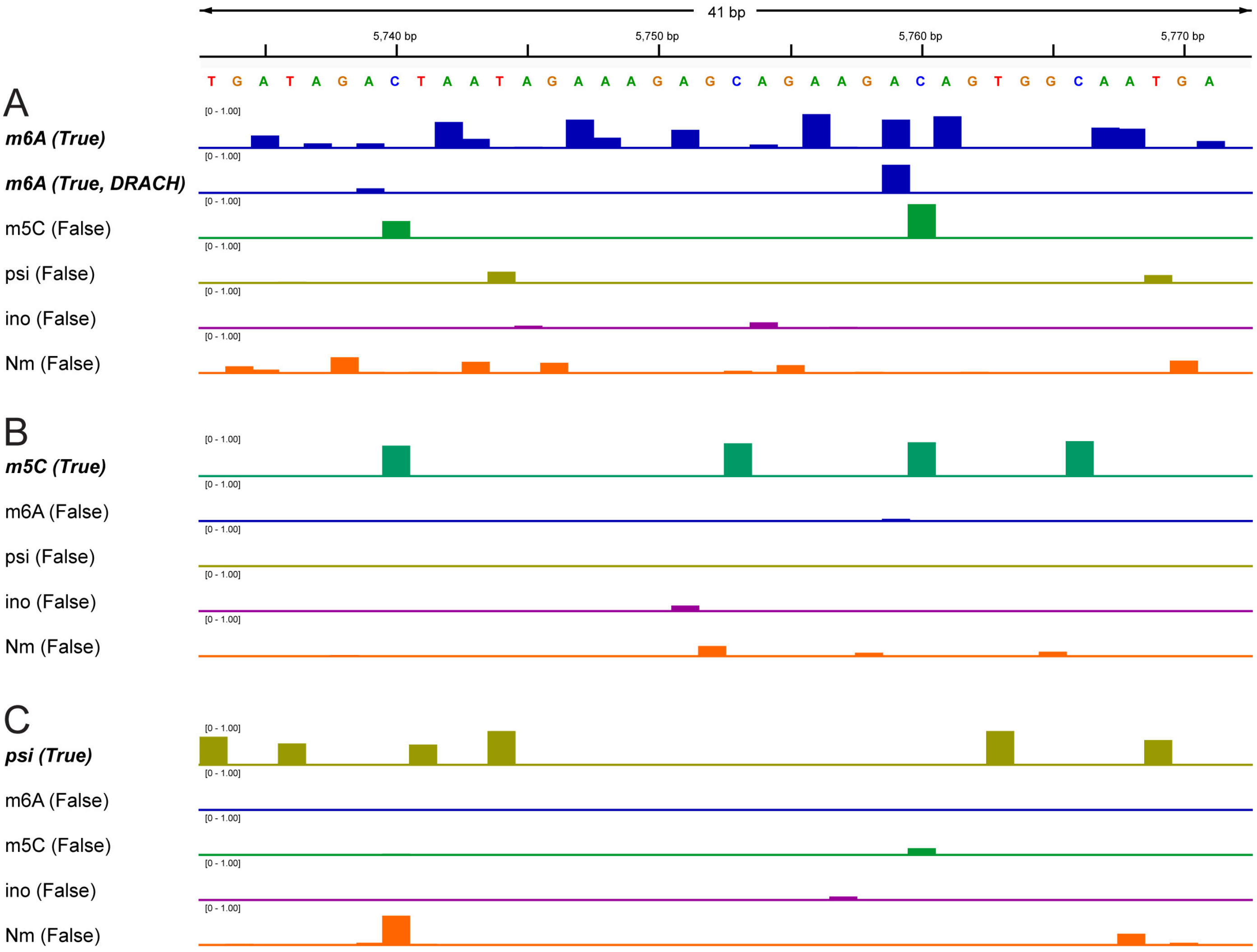

<u>Supplementary Fig. S6</u>. Miscalled modifications due to adjacent modifications. X-axis of each panel is the nucleotide position that corresponds to the sequence at the top of the figure. Y-axis is the percentage of reads containing a modification that position, according to Nanopore RNA modification-calling algorithm. m^{6}A (blue), m^{5}C (green), psi (yellow), inosine (purple), and 2'-*O*-Me (orange). (**A**) is derived from T7 *in vitro* transcribed RNA in which all As have been replaced with m6A. (**B**) is derived from T7 *in vitro* transcribed RNA in which all Cs have been replaced with m5C. (**C**) is derived from T7 *in vitro* transcribed RNA in which all Us have been replaced with psi.

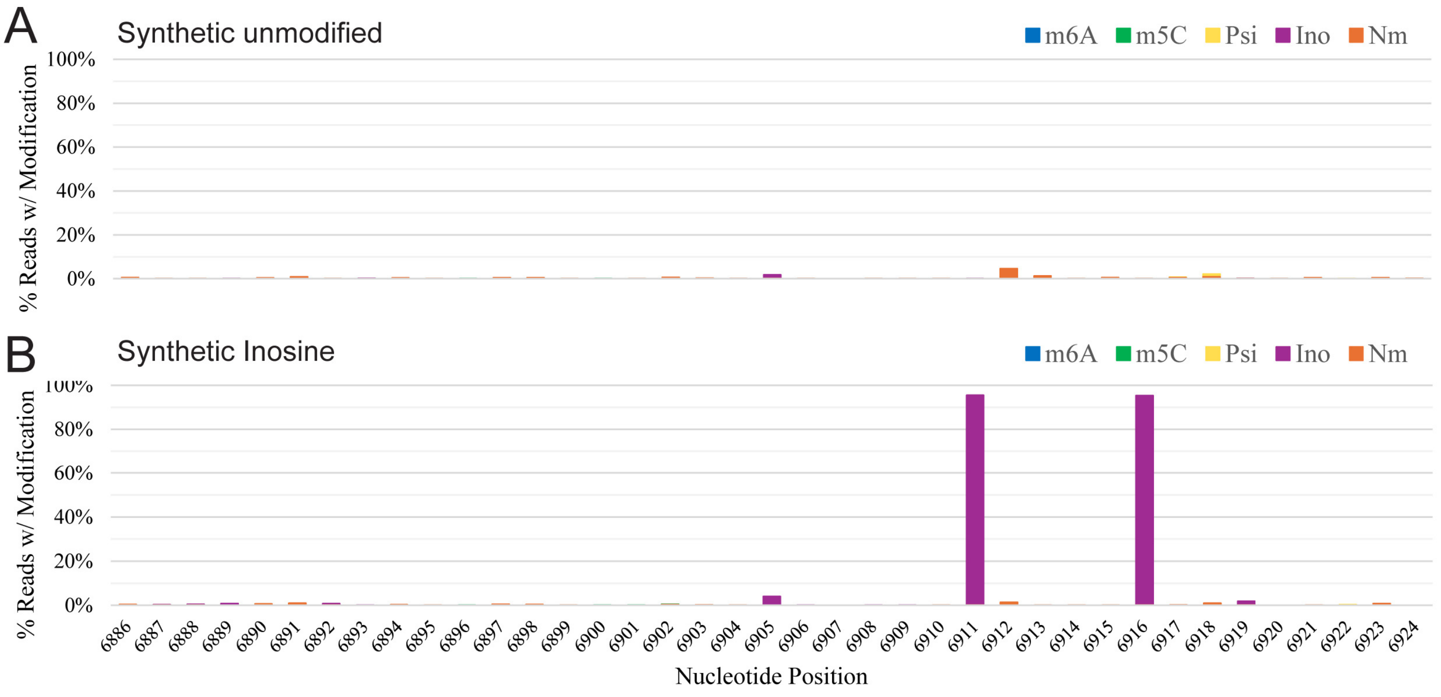

Supplementary Fig. S7. Inosine calling in synthetic RNA fragments. Comparison of modification calling between two synthetic HIV-1 RNA fragments, one unmodified (**A**) and one bearing inosine (**B**) at two suspected inosine locations based on reference sequencing data. The nucleotide position corresponding to the NL4-3 genome is indicated on the x-axis. Synthesized fragments were generated based on the reverse strand of the NL4-3 genome.

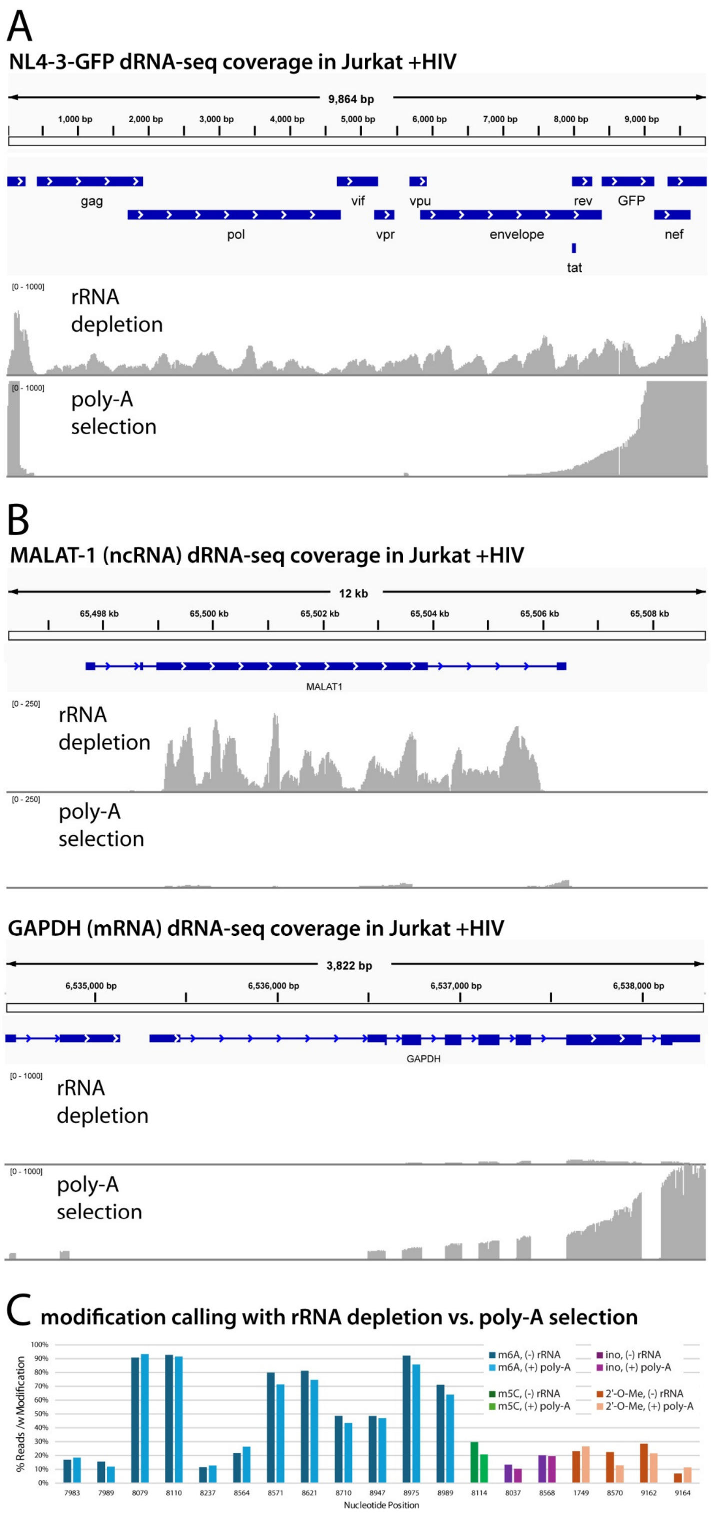

D

RNA type	Poly-A	RNaseH
mRNA	95.3%	35.3%
snRNA	0.0%	21.0%
lncRNA	2.2%	18.0%
miRNA	0.0%	11.3%
misc RNA	0.0%	10.9%
snoRNA	0.0%	2.8%
rRNA	0.0%	0.1%
Mt rRNA	2.0%	0.0%

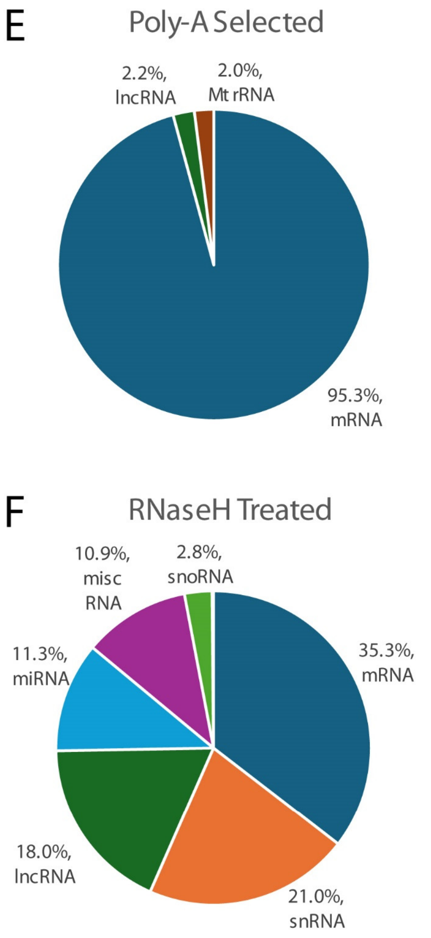

Supplementary Fig. S8. **Comparison of rRNA depletion and poly-A selection using read coverage maps and modified base calling from nanopore dRNA-seq**. X-axis is the individual nucleotide positions. Y-axis is the number of reads with coverage at that position. Samples are from Jurkat cells infected with NL4-3-GFP HIV-1. (**A**) Comparison of HIV-1 sequencing coverage from samples prepared by rRNA depletion or poly-A selection. (**B**) Comparison of human noncoding RNA MALAT-1 and mRNA GAPDH sequencing coverage for each RNA preparation method. (**C**) Modification calling results for HIV-1 viral RNA taken from Jurkat cell cultures and either poly-A selected or rRNA depleted prior to dRNA-seq. m^{6}A (blue), m^{5}C (green), pseudouridine (psi) (yellow), inosine (purple) and 2'-*O*-methylation (orange). (**D-F**) Table and pie charts comparing RNA type distribution for sequenced samples taken from poly-A selected or rRNA depleted RNA. Counts are derived from Salmon TPM based on alignment of samples against the human transcriptome.

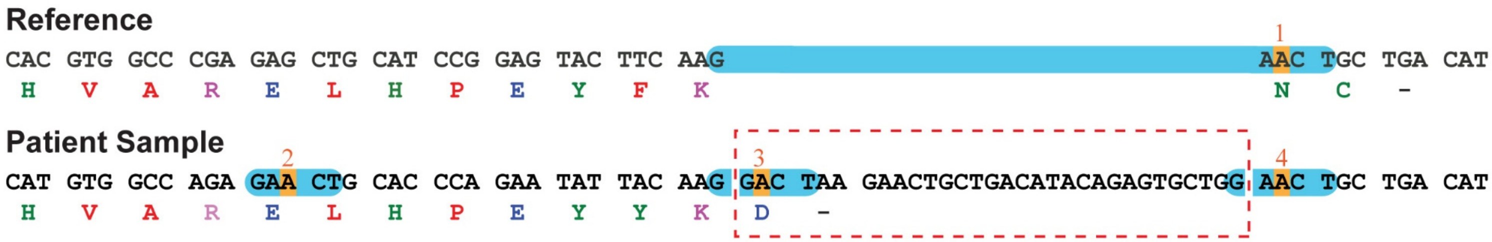

<u>Supplementary Fig. S9</u>. Additional DRACH motif sites identified in a patient sample. Comparison of sequence and m^{6}A modifications between a sample with reference HIV sequence NL4-3-GFP (7C) and a PLWH sample (P8). The region numbered 8910-8990 is shown, which corresponds to the 3' end of the *nef* gene and the 3' LTR. The P8 patient sample has two insertions and several SNPs compared to the reference sequence, which introduces new DRACH motif and changes the stop codon of the gene. DRACH motif sites are marked in blue, with the A which is modified to m^{6}A in orange. STOP codons are marked with a dash in the amino acid sequence. Patient DRACH motif site 2 is the result of a SNP which changes G to A. The existing DRACH motif in the reference sequence (1) is the site of an insertion, resulting in patient sample DRACH motif sites 3 and 4. Despite the large insert, only one additional amino acid is added to the nef protein due to a stop codon in the insertion.

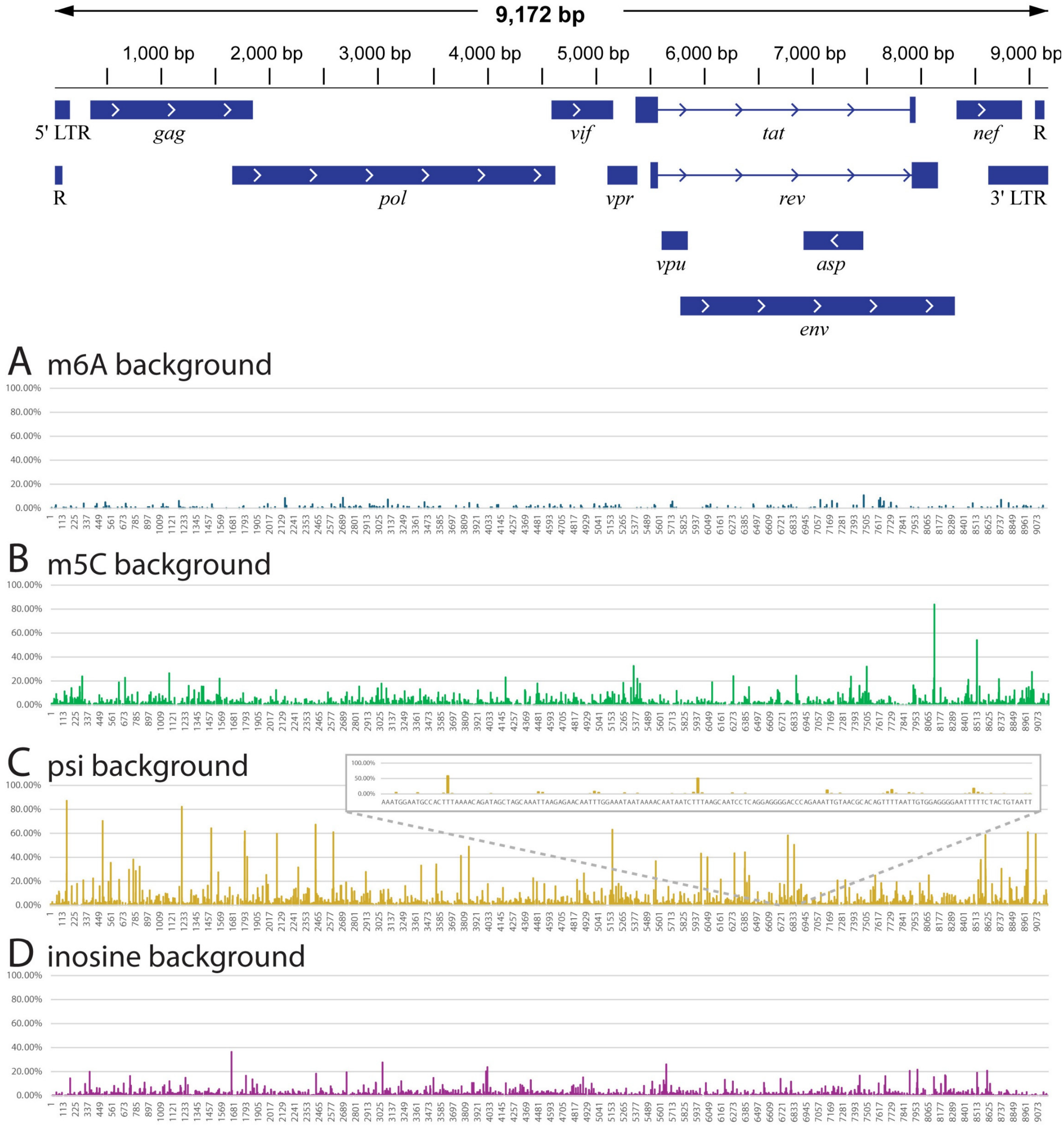

<u>Supplementary Fig. S10</u>. Modification calling from nanopore sequencing of *in vitro* transcribed NL4-3 HIV-1 RNA fragments used for baseline correction, using older Nanopore modification calling algorithm. Utilizes Dorado RNA modification basecalling algorithm rna004_130bps_sup@v5.1.0. X-axis represents individual nucleotides in the HIV-1 genome. Y-axis represents percentage of reads that contained the mutation of interest for that nucleotide. (**A**) m^6A (blue), (**B**) m^5C (green), (**C**) pseudouridine (psi) (yellow), (**D**) inosine (purple). Dorado algorithm rna004_130bps_sup@v5.1.0 does not support 2'-*O*-methylation calling.
