# Supplementary material for: A nanopore-based HIV-1 reference epitranscriptome
Source: Nucleic Acids Res. 2026 Mar 19;54(6):gkag220. doi: 10.1093/nar/gkag220 (PMC13000461; doi:10.1093/nar/gkag220)
Supplement: gkag220_Supplemental_Files [file gkag220_supplemental_files.zip › Bosmeny_HIV1-Ref-epi_SuppTables_revised.docx]

# **Supplementary Table S1. DNA oligonucleotides used in this study.**

| **Name** | **Sequence(5'->3')** | **Notes** |
| --- | --- | --- |
| NL4-3_Seg1_F_T7 | TAATACGACTCACTATAgggagaagtattagtgtggaag | Generates 3452nt PCR fragment for sense |
| NL4-3_Seg1_R | GGGTCATAATACACTCCATGTACC | transcription of NL4-3 from 1 to 3048nt. |
| NL4-3_Seg1_F | gggagaagtattagtgtggaag | Generates 3452nt PCR fragment for antisense |
| NL4-3_Seg1_R_T7 | TAATACGACTCACTATAGGGTCATAATACACTCCATGTACC | transcription of NL4-3 from 1 to 3048nt. |
| NL4-3_Seg2_F_T7 | TAATACGACTCACTATAGGGAAGTTCAATTAGGAATACCAC | Generates 3065nt PCR fragment for sense |
| NL4-3_Seg2_R | GGGCTCTAGTCTAGGATCTAC | transcription of NL4-3 from 2359 to 5406nt. |
| NL4-3_Seg2_F | GGGAAGTTCAATTAGGAATACCAC | Generates 3065nt PCR fragment for antisense |
| NL4-3_Seg2_R_T7 | TAATACGACTCACTATAGGGCTCTAGTCTAGGATCTAC | transcription of NL4-3 from 2359 to 5406nt. |
| NL4-3_Seg3_F_T7 | TAATACGACTCACTATAGGGAGTCTCCATAGAATGGAG | Generates 3101nt PCR fragment for sense |
| NL4-3_Seg3_R | GGGTCTGAAACGATAATGGTG | transcription of NL4-3 from 4836 to nt. |
| NL4-3_Seg3_F | GGGAGTCTCCATAGAATGGAG | Generates 3101nt PCR fragment for antisense |
| NL4-3_Seg3_R_T7 | TAATACGACTCACTATAGGGTCTGAAACGATAATGGTG | transcription of NL4-3 from 4836 to 7919nt. |
| NL4-3_Seg4_F_T7 | TAATACGACTCACTATAGGGCATCAAACAGCTCC | Generates 1747nt PCR fragment for sense |
| NL4-3_Seg4_R | gggatctctagttaccagagtc | transcription of NL4-3 from 7474 to 9172nt. |
| NL4-3_Seg4_F | GGGCATCAAACAGCTCC | Generates 1747nt PCR fragment for antisense |
| NL4-3_Seg4_R_T7 | TAATACGACTCACTATAgggatctctagttaccagagtc | transcription of NL4-3 from 7474 to 9172nt. |
|  |  |  |
| NL520R | TAAGCAGTGGGTTCCCTAGT | Primers from Baek et al. (2024) |
| NL590R | GAGGGATCTCTAGTTACCAG | for improved RT during sequencing prep |
| NL660R | CTTTCAAGTCCCTGTTCGGG |  |
| NL780R | TCTAGCCTCCGCTAGTCAAA |  |
| NL870R | TTTCTTTCCCCCTGGCCTTA |  |
| NL930R | AAAGGCCAGGATTAACTGCG |  |
| NL1010R | TCTTCTGATCCTGTCTGAAG |  |
| NL1097R | TTATCTAAGGCTTCCTTGGTGTC |  |
| NL1160R | TTTCCTGTGTCAGCTGCTGC |  |
| NL1240R | TTCTAGGTGATATGGCCTGA |  |
| NL1290R | TTCTGGGCTGAAAGCCTTCT |  |
| NL1380R | GGCTGCTTGATGTCCCCCCA |  |
| NL1460R | GCAATAGGCCCTGCATGCAC |  |
| NL1550R | GGATTATGTGTCATCCATCC |  |
| NL1640R | TCCAGAATGCTGGTAGGGCT |  |
| NL1720R | GTGAAGCTTGCTCGGCTCTT |  |
| NL1820R | TCTAGTGTCGCTCCTGGTCC |  |
| NL1910R | GCTGGATTTGTTACTTGGCT |  |
| NL2010R | GGCCCTGCAATTTTTGGCTA |  |
| NL2120R | CTTCCCTTGTGGGAAGGCCA |  |
| NL2210R | TGAGAGGGAGTTGTTGTCTC |  |
| NL2280R | GGGGTCGCTGCCAAAGAGTG |  |
| NL2370R | CTGGCAAATTCATTTCTTCT |  |
| NL2470R | CCTATAGCTTTATGTCCGCA |  |
| NL2550R | GAAAATTTAAAGTGCAGCCA |  |
| NL2610R | CTTTTGGGCCATCCATTCCT |  |
| NL2710R | ATTGTATGGATTTTCAGGCC |  |
| NL2800R | GAACTTCCCAGAAATCTTGA |  |
| NL2890R | GCATCGCCCACATCCAGTAC |  |
| NL2980R | TATCTAATCCCTGGTGTCTC |  |
| NL3090R | AGATGACTATGTCTGGATTT |  |
| NL3180R | TCAACAGATGTTGTCTCAGT |  |
| NL3270R | CTGTCCATTTATCAGGATGG |  |
| NL3350R | TGCCCAATTCAATTTTCCCA |  |
| NL3420R | CTGTTAGTGCTTTGGTTCCC |  |
| NL3500R | CACTCCATGTACCGGTTCTTTTAG |  |
| NL3570R | TTTGATATGTCCATTGGCCT |  |
| NL3660R | CCTCTGTTAATTGTTTCACA |  |
| NL3760R | TCTGTCCACCATGCTTCCCA |  |
| NL3840R | GTACCATAACTTCACTAAGG |  |
| NL3890R | ATTGGCTGCCCCATCTACAT |  |
| NL3940R | CCGTTAGGGGGACAACTTTT |  |
| NL4060R | CCCAATGCATATTGTGAGTC |  |
| NL4170R | CTTTGTGTGCTGGTACCCAT |  |
| NL4250R | CTTATCTATTCCATCTAAAA |  |
| NL4320R | CAGGTGGTAGGTTAAAATCA |  |
| NL4400R | GCTACAGTCTACTTGTCCAT |  |
| NL4500R | CTGGAATTACTTCTGCTTCT |  |
| NL4600R | GTACTGGTGAAATTGCTGCC |  |
| NL4680R | TTACTCCTTGACTTTGGGGATTG |  |
| NL4760R | TTGTACTGCTGTCTTAAGAT |  |
| NL4850R | GTCTGTTGCTATTATGTCTA |  |
| NL4920R | TGCTGTCCCTGTAATAAACC |  |
| NL4990R | CTCATCCTGTCTACTTGCCA |  |
| NL5030R | GATCTTTGCTTTTCTTCTTGGCACTACT |  |
| NL5180R | GTACTTTCATAGTGATGTCT |  |
| NL5270R | CTTTCTCCTGTATGCAGACC |  |
| NL5360R | ATTAGTTGGTCTGCTAGGTC |  |
| NL5440R | CACACCTAGGACTAACTATA |  |
| NL5540R | CTAACACTAGGCAAAGGTGG |  |
| NL5610R | ATTCATTGTATGGCTCCCTCTG |  |
| NL5720R | CCAAGTATCCCCGTAAGTTT |  |
| NL5820R | TCCTCTGTCGAGTAACGCCT |  |
| NL5880R | CTGACTTCCTGGATGCTTCC |  |
| NL5940R | ACAAACTTGGCAATGAAAGC |  |
| NL6020R | GTCTGACTGTTCTGATGAGC |  |
| NL6130R | ATGATTACTATGGACCACAC |  |
| NL6214R | TGTCTTCTGCTCTTTCTATTAGTCTA |  |
| NL6300R | ATCATCAATATCCCAAGGAG |  |
| NL6400R | TGCTTTAGCATCTGATGCAC |  |
| NL6510R | TTCCACATGTTAAAATTTTCTGTCACATT |  |
| NL6590R | GGGTTAATTTTACACATGGC |  |
| NL6660R | ATTCTCCCGCTACTACTATT |  |
| NL6740R | CATATTCTTTCTGCACCTTA |  |
| NL6800R | AACTTATCAACCTATAGCTG |  |
| NL6880R | ACCAGCCGGGGCACAATAAT |  |
| NL6950R | GTACTGTGCTGACATTTGTA |  |
| NL7020R | TCTTCTTCTGCTAGACTGCC |  |
| NL7112R | TGGGTCTTGTACAATTAATTTCTACAGA |  |
| NL7200R | GCTTGTCTCATATTTCCTAT |  |
| NL7310R | CTGAGGATTGCTTAAAGATT |  |
| NL7370R | TACAGTAGAAAAATTCCCCT |  |
| NL7440R | TCAGTGTTATTTGACCCTTC |  |
| NL7500R | TCCTGCCACATGTTTATAAA |  |
| NL7580R | TTGTTAATAGCAGCCCAGTA |  |
| NL7660R | TTCACTTCTCCAATTGTCCC |  |
| NL7740R | TCTCTCTGCACCACTCTTCT |  |
| NL7820R | TCATTGACGCTGCGCCCATA |  |
| NL7910R | ACAGATGCTGTTGCGCCTCA |  |
| NL7970R | ATCTTTCCACAGCCAGGATT |  |
| NL8040R | GGCACAGCAGTGGTGCAAAT |  |
| NL8120R | TAATTTCTCTGTCCCACTCC |  |
| NL8180R | TCTTTTCTTGCTGGTTTTGC |  |
| NL8380R | TGGGAGGTGGGTCTGAAACG |  |
| NL8450R | TGGATCTGTCTCTGTCTCTC |  |
| NL8520R | TGGTAGCTGAAGAGGCACAG |  |
| NL8600R | AATATTTGAGGGCTTCCCAC |  |
| NL8680R | TACTGCTATGGCTGTGGCAT |  |
| NL8750R | TTCTAGGTATGTGGCGAATA |  |
| NL8810R | ACTTTTTGACCACTTGCCAC |  |
| NL8894R | TACTTGTGATTGCTCCATGTTTTTCTAGG |  |
| NL8980R | TCTTCCTCCTCTTGTGCTTC |  |
| NL9050R | GCTAAGATCTACAGCTGCCT |  |
| NL9140R | TTGTGTGTGGTAGATCCACA |  |
| NL9220R | AGCTTGTAGCACCATCCAAA |  |
| NL9290R | CACAGGGTGTAACAAGCTGG |  |
| NL9360R | GAAATGCTAGGCGGCTGTCA |  |
| NL9530R | CCAGTACAGGCAAAAAGCAG |  |
| NL9600R | AGGCTTAAGCAGTGGGTTCC |  |
| NL9660R | ACCAGAGTCACACAACAGAC |  |
|  |  |  |
| AG9454_28_90 | CCGCCCCCTCCGGGGAGGAGGAGGAGGGGCGGCGGGGGAAGGGAGGGCGG | Oligomers used in enzymatic depletion |
| AG9405_28_41 | GGGCGCGGGGTGGGGAGGGAGCGAGCGGCGCGCGCGGGTGGGGCGGGGGA | of ribosomal RNA |
| AG9443_28_79 | GCACCCCCCCCGTCGCCGGGGCGGGGGCGCGGGGAGGAGGGGTGGGAGAG |  |
| AG9449_28_85 | CCCGACCCGCGCGCCCTCCCGAGGGAGGACGCGGGGCCGGGGGGCGGAGA |  |
| AG9421_28_57 | GGGCGGGGAGCGGGGCGTGGGCGGGAGGAGGGGAGGAGGCGTGGG |  |
| AG9447_28_83 | GGGATTCGGCGAGTGCTGCTGCCGGGGGGGCTGTAACACTCGGGGIGGGT |  |
| AG9396_28_32 | GCCGCCGCCGCCGCCGCGCGCCGAGGAGGAGGGGGGAACGGGGGGCGGAC |  |
| AG9404_28_40 | CTCCCCCGGGGAGGGGGGAGGACGGGGAGCGGGGGAGAGAGAGAGAGAGA |  |
| AG9514_12_15 | CGCGGTGGCTGGCACGAAATTGACCAACCCTGGGGTTAGTATAGCTTAGT |  |
| AG9349_18_23 | TAAGAGCATCGAGGGGGCGCCGAGAGGCAAGGGGCGGGGACGGGCGGTGG |  |
| AG9398_28_34 | CCGACCGCCGCCGCCCGACCGCTCCCGCCCCCAGCGGACGCGCGCGCGAC |  |
| AG9370_28_6 | GGGCGCGTGGAGGGGIGGGCGGCCCGCCGGCGGGGACAGGCGGGGGACCG |  |
| AG9373_28_9 | TTCGCCCCATTGGCTCCTCAGCCAAGCACATACACCAAATGTCTGAACCT |  |
| AG9452_28_88 | GGTGGAAATGCGCCCGGCGGCGGCCGGTCGCCGGTCGGGGGACGGTCCCC |  |
| AG9346_18_20 | CTTAATCATGGCCTCAGTTCCGAAAACCAACAAAATAGAACCGCGGTCCT |  |
| AG9430_28_66 | CCATTTAAAGTTTGAGAATAGGTTGAGATCGTTTCGGCCCCAAGACCTCT |  |
| AG9395_28_31 | CCGGGGGCGGACCCGGCGGGIGGGACCGGCCCGCGGCCCCTCCGCCGCCT |  |
| AG9450_28_86 | CGGGGGAGGAGGAGGACGGACGGACGGACGGGGCCCCCCGAGCCACCTTC |  |
| AG9501_12_2 | CTCTATATAAATGCGTAGGGGTTTTAGTTAAATGTCCTTTGAAGTATACT |  |
| AG9335_18_9 | GGTGGCTGAACGCCACTTGTCCCTCTAAGAAGTTGGGGGACGCCGACCGC |  |
| AG9482_16_14 | GGGTACCGCGGCCGTTAAACATGTGTCACTGGGCAGGCGGTGCCTCTAAT |  |
| AG9406_28_42 | GGGCCGCGAGGGGGGTGCCCCGGGCGTGGGGIGGGCGCGCGCCTCGTCCA |  |
| AG9399_28_35 | CGAGACGTGGGGTGGGGGTGGGGGGCGCGCCGCGCCGCCGCCGGGCTCCC |  |
| AG9365_28_1 | GACAAACCCTTGTGTCGAGGGCTGACTTTCAATAGATCGCAGCGAGGGAG |  |
| AG9361_18_35 | TTAGCTCTAGAATTACCACAGTTATCCAAGTAGGAGAGGAGCGAGCGACC |  |
| AG9389_28_25 | TGGATAGTAGGTAGGGACAGTGGGAATCTCGTTCATCCATTCATGCGCGT |  |
| AG9384_28_20 | CCCTCGGGCTCGCCCCCCCGCCTCACCGGGTCAGTGAAAAAACGATCAGA |  |
| AG9372_28_8 | GGCGGGATTCTGACTTAGAGGCGTTCAGTCATAATCCCACAGATGGTAGC |  |
| AG9383_28_19 | GGAGCGGGTCGCGCCCGGCCGGGCGGGCGCTTGGCGCCAGAAGCGAGAGC |  |
| AG9333_18_7 | CCTGCCGGCGTAGGGTAGGCACACGCTGAGCCAGTCAGTGTAGCGCGCGT |  |
| AG9444_28_80 | CGGTCGCGCCGTGGGAGGGGTGGCCCGGCCCCCCCACGAGGAGACGCCGG |  |
| AG9350_18_24 | CTCGCCTCGCGGCGGACCGCCCGCCCGCTCCCAAGATCCAACTACGAGCT |  |
| AG9445_28_81 | CGCGCCCCCGCGGGGGAGACCCCCCTCGCGGGGGATTCCCCGCGGGGGTG |  |
| AG9341_18_15 | CTCCCCCCGGAACCCAAAGACTTTGGTTTCCCGGAAGCTGCCCGGCGGGT |  |
| AG9456_28_92 | ACACGGCCGGACCCGCCGCCGGGTTGAATCCTCCGGGCGGACTGCGCGGA |  |
| AG9441_28_77 | GACGCGCGCGTGGCCCCGAGAGAACCTCCCCCGGGCCCGACGGCGCGACC |  |
| AG9327_18_1 | TAATGATCCTTCCGCAGGTTCACCTACGGAAACCTTGTTACGACTTTTAC |  |
| AG9344_18_18 | ATGAAAACATTCTTGGCAAATGCTTTCGCTCTGGTCCGTCTTGCGCCGGT |  |
| AG9400_28_36 | CGGGGGCGGCCGCGACGCCCGCCGCAGCTGGGGCGATCCACGGGAAGGGC |  |
| AG9512_12_13 | GAGTTTTTTACAACTCAGGTGAGTTTTAGCTTTATTGGGGAGGGGGTGAT |  |
| AG9397_28_33 | GGGCCGGGIGGGTAGGGCGGGGGGACGAACCGCCCCGCCCCGCCGCCCG |  |
| AG9401_28_37 | CCGGCTCGCGTCCAGAGTCCGCGCCGCCGCCGGCCCCCCGGGTCCCCGGG |  |
| AG9359_18_33 | GCCCCCCGGCCGGGGCCGGAGAGGGGCTGACCGGGTTGGTTTTGATCTGA |  |
| AG9431_28_67 | AATCATTCGCTTTACCGGATAAAACTGCGTGGCGGGGGTGCGTCGGGTCT |  |
| AG9466_5.8_1 | AAGCGACGCTCAGACAGGCGTAGCCCCGGGAGGAACCCGGGGCCGCAAGT |  |
| AG9422_28_58 | GGGCGGGGGAAGGACCCCACACCCCCGCCGCCGCCGCCGCCGCCGCCCTC |  |
| AG9394_28_30 | AGTCGGCTGCTAGGCGCCGGCCGAGGCGAGGCGCGCGCGGAACCGCGGCC |  |
| AG9469_16_1 | AAACCCTGTTCTTGGGTGGGTGTGGGTATAATACTAAGTTGAGATGATAT |  |
| AG9439_28_75 | ACGTGTTAGACTCCTTGGTCCGTGTTTCAAGACGGGTCGGGTGGGTAGCC |  |
| AG9436_28_72 | CTCCACCTCCCCGGCGCGGCGGGCGAGACGGGCCGGTGGTGCGCCCTCGG |  |
| AG9499_16_31 | AATGGTTTGGCTAAGGTTGTCTGGTAGTAAGGTGGAGTGGGTTTGGGGCT |  |
| AG9461_28_97 | GCCTCACACCGTCCACGGGCTGGGCCTCGATCAGAAGGACTTGGGCCCCC |  |
| AG9446_28_82 | GGCGCCGGGAGGGGGGAGAGCGCGGCGACGGGTCTCGCTCCCTCGGCCCC |  |
| AG9329_18_3 | GTGGGCCGACCCCGGCGGGGCCGATCCGAGGGCCTCACTAAACCATCCAA |  |
| AG9357_18_31 | TCGAAAGTTGATAGGGCAGACGTTCGAATGGGTCGTCGCCGCCACGGG |  |
| AG9352_18_26 | GCTGCTGGCACCAGACTTGCCCTCCAATGGATCCTCGTTAAAGGATTTAA |  |
| AG9455_28_91 | GTGGAGGGGTCGGGAGGAACGGGGGGCGGGAAAGATCCGCCGGGCCGCCG |  |
| AG9374_28_10 | GCGGTTCCTCTCGTACTGAGCAGGATTACCATGGCAACAACACATCATCA |  |
| AG9386_28_22 | CCCTCGCGGGGACACCGGGIGGGCGCCGGGGGCCTCCCACTTATTCTACA |  |
| AG9448_28_84 | TTCGGTCCCGCCGCCCCCGCCGCCGCCGCCACCGCCGCCGCCGCCGCCGC |  |
| AG9418_28_54 | ACTTCGGCCTTCAAAGTTCTCGTTTGAATATTTGCTACTACCACCAAGAT |  |
| AG9432_28_68 | GCGAGAGCGCCAGCTATCCTGAGGGAAACTTCGGAGGGAACCAGCTACTA |  |
| AG9518_12_19 | CTTGCATGTGTAATCTTACTAAGAGCTAATAGAAAGGCTAGGACCAAACC |  |
| AG9351_18_25 | TTTTAACTGCAGCAACTTTAATATACGCTATTGGAGCTGGAATTACCGCG |  |
| AG9509_12_10 | GGCTCGTAGTGTTCTGGCGAGCAGTTTTGTTGATTTAACTGTTGAGGTTT |  |
| AG9336_18_10 | TCGGGGGTCGCGTAACTAGTTAGCATGCCAGAGTCTCGTTCGTTATCGGA |  |
| AG9367_28_3 | GGTTTAGCGCCAGGTTCCCCACGAACGTGCGGTGCGTGACGGGCGAGGG |  |
| AG9328_18_2 | TTCCTCTAGATAGTCAAGTTCGACCGTCTTCTCAGCGCTCCGCCAGGGCC |  |
| AG9437_28_73 | CGGACTGGAGAGGCCTCGGGATCCCACCTCGGCCGGCGAGCGCGCCGGCC |  |
| AG9381_28_17 | ACGGGAGGTTTCTGTCCTCCCTGAGCTCGCCTTAGGACACCTGCGTTACC |  |
| AG9345_18_19 | CCAAGAATTTCACCTCTAGCGGCGCAATACGAATGCCCCCGGCCGTCCCT |  |
| AG9348_18_22 | CACTCTAATTTTTTCAAAGTAAACGCTTCGGGCCCCGCGGGACACTCAGC |  |
| AG9471_16_3 | TTCGTACAGGGAGGAATTTGAANGTAGATAGAAACCGACCTGGATTACTC |  |
| AG9385_28_21 | GTAGTGGTATTTCACCGGCGGCCCGCAGGGCCGCGGACCCCGCCCCGGGC |  |
| AG9438_28_74 | TTCACCTTCATTGCGCCACGGCGGCTTTCGTGCGAGCCCCCGACTCGCGC |  |
| AG9470_16_2 | CATTTACGGGGGAAGGCGCTTTGTGAAGTAGGCCTTATTTCTCTTGTCCT |  |
| AG9363_18_37 | CCGTGCGTACTTAGACATGCATGGCTTAATCTTTGAGACAAGCATATGCT |  |
| AG9521_5_3 | TTCCGAGATCAGACGAGATCGGGCGCGTTCAGGGTGGTATGGCCGTAGAC |  |
| AG9493_16_25 | TCTTGGACAACCAGCTATCACCAGGCTCGGTAGGTTTGTCGCCTCTACCT |  |
| AG9355_18_29 | CCTTCCTTGGATGTGGTAGCCGTTTCTCAGGCTCCCTCTCCGGAATCGAA |  |
| AG9520_5_2 | CCAGGCCCGACCCTGCTTAGCTTCCGAGATCAGACGAGATCGGGCGCGTT |  |
| AG9382_28_18 | GTTTGACAGGTGTACCGCCCCAGTCAAACTCCCCACCTGGCACTGTCCCC |  |
| AG9358_18_32 | GCGTGCGATCGGCCCGAGGTTATCTAGAGTCACCAAAGCCGCCGGCGCCC |  |
| AG9362_18_36 | AAAGGAACCATAACTGATTTAATGAGCCATTCGCAGTTTCACTGTACCGG |  |
| AG9413_28_49 | CTGCCCTTCACAAAGAAAAGAGAACTCTCCCCGGGGCTCCCGCCGGCTTC |  |
| AG9451_28_87 | CCCGCCGGGCCTTCCCAGCCGTCCCGGAGCCGGTCGCGGCGCACCGCCGC |  |
| AG9337_18_11 | ATTAACCAGACAAATCGCTCCACCAACTAAGAACGGCCATGCACCACCAC |  |
| AG9407_28_43 | GCCGCGGCGCGCGCCCAGCCCCGCTTCGCGCCCCAGCCCGACCGACCCAG |  |
| AG9506_12_7 | CTTACTTTGTAGCCTTCATCAGGGTTTGCTGAAGATGGCGGTATATAGGC |  |
| AG9425_28_61 | ATCCATTTTCAGGGCTAGTTGATTCGGCAGGTGAGTTGTTACACACTCCT |  |
| AG9502_12_3 | TGAGGAGGGTGACGGGCGGTGTGTACGCGCTTCAGGGCCCTGTTCAACTA |  |
| AG9360_18_34 | TAAATGCACGCATCCCCCCCGCGAAGGGGGTCAGCGCCCGTCGGCATGTA |  |
| AG9420_28_56 | ACCGCAGCGGCCCTCCTACTCGTCGCGGCGTAGCGTCCGCGGGGCTCCGG |  |
| AG9460_28_96 | CCCAAGCAACCCGACTCCGGGAAGACCCGGGCGCGCGCCGGCCGCTACCG |  |
| AG9368_28_4 | GCGGCCGCCTTTCCGGCCGCGCCCCGTTTCCCAGGACGAAGGGCACTCCG |  |
| AG9489_16_21 | TTTAGGTAGTGGGTGTTGAGCTTGAACGCTTTCTTAATTGGTGGCTGCTT |  |
| AG9402_28_38 | GCCCCCCTCGCGGGGACCTGCCCCCGCCGGCCGCCCCGGCGGCCGCCGCG |  |
| AG9486_16_18 | GGCTGTTAATTGTCAGTTCAGTGTTTTAATCTGACGCAGGCTTATGCGGA |  |
| AG9364_18_38 | TGGCTTAATCTTTGAGACAAGCATATGCTACTGGCAGGATCAACCAGGTA |  |
| AG9513_12_14 | CTAAAACACTCTTTACGCCGGCTTCTATTGACTTGGGTTAATCGTGTGAC |  |
| AG9377_28_13 | ATAGGAAGAGCCGACATCGAAGGATCAAAAAGCGACGTCGCTATGAACGC |  |
| AG9412_28_48 | CTTTCCAAGGCACGGGCCCCTCTCTCGGGGCGAACCCATTCCAGGGCGCC |  |
| AG9440_28_76 | GACGTCGCCGCCGACCCCGTGCGCTCGCTCCGCCGTCCCCCTCTTCGGG |  |
| AG9490_16_22 | TTAGGCCTACTATGGGTGTTAAATTTTTTACTCTCTCTACAAGGTTTTTT |  |
| AG9508_12_9 | TCCTCTAGAGGGATATGAAGCACCGCCAGGTCCTTTGAGTTTTAAGCTGT |  |
| AG9409_28_45 | CCCTTACCTACATTGTTCCAACATGCCAGAGGCTGTTCACCTTGGAGACC |  |
| AG9414_28_50 | TCCGGGATCGGTCGCGTTACCGCACTGGACGCCTCGCGGCGCCCATCTCC |  |
| AG9462_28_98 | CACGAGCGGCGCCGGGGAGCGGGTCTTCCGTACGCCACATGTCCCGCGCC |  |
| AG9330_18_4 | TCGGTAGTAGCGACGGGCGGTGTGTACAAAGGGCAGGGACTTAATCAACG |  |
| AG9487_16_19 | GGAGAATGTTTTCATGTTACTTATACTAACATTAGTTCTTCTATAGGGTG |  |
| AG9428_28_64 | GTTCGGTTCATCCCGCAGCGCCAGTTCTGCTTACCAAAAGTGGCCCACTA |  |
| AG9498_16_30 | TATCTATTGCGCCAGGTTTCAATTTCTATCGCCTATACTTTATTTGGGTA |  |
| AG9366_28_2 | CTGCTCTGCTACGTACGAAACCCCGACCCAGAAGCAGGTCGTCTACGAAT |  |
| AG9332_18_6 | ATTGCAATCCCCGATCCCCATCACGAATGGGGTTCAACGGGTTACCCGCG |  |
| AG9356_18_30 | CCCTGATTCCCCGTCACCCGTGGTCACCATGGTAGGCACGGCGACTACCA |  |
| AG9459_28_95 | TGCCGGTATTTAGCCTTAGATGGAGTTTACCACCCGCTTTGGGCTGCATT |  |
| AG9479_16_11 | AGGGTCTTCTCGTCTTGCTGTGTTATGCCCGCCTCTTCACGGGCAGGTCA |  |
| AG9480_16_12 | ATTTCACTGGTTAAAAGTAAGAGACAGCTGAACCCTCGTGGAGCCATTCA |  |
| AG9503_12_4 | AGCACTCTACTCTTAGTTTACTGCTAAATCCACCTTCGACCCTTAAGTTT |  |
| AG9464_28_100 | CTGAGGGAATCCTGGTTAGTTTCTTTTCCTCCGCTGACTAATATGCTTAA |  |
| AG9457_28_93 | CCCCACCCGTTTACCTCTTAACGGTTTCACGCCCTCTTGAACTCTCTCTT |  |
| AG9475_16_7 | GGGTAACTTGTTCCGTTGGTCAAGTTATTGGATCAATTGAGTATAGTAGT |  |
| AG9369_28_5 | CACCGGACCCCGGTCCCGGCGCGCGGCGGGGCACGCGCCCTCCCGCGGCG |  |
| AG9434_28_70 | GCACGTCAGGACCGCTACGGACCTCCACCAGAGTTTCCTCTGGCTTCGCC |  |
| AG9408_28_44 | CCCTTAGAGCCAATCCTTATCCCGAAGTTACGGATCCGGCTTGCCGACTT |  |
| AG9416_28_52 | GGCAACGGAGGCCATCGCCCGTCCCTTCGGAACGGCGCTCGCCCATCTCT |  |
| AG9507_12_8 | TGAGCAAGAGGTGGTGAGGTTGATCGGGGTTTATCGATTACAGAACAGGC |  |
| AG9354_18_28 | TTTCGTCACTACCTCCCCGGGTCGGGAGTGGGTAATTTGCGCGCCTGCTG |  |
| AG9353_18_27 | AGTGGACTCATTCCAATTACAGGGCCTCGAAAGAGTCCTGTATTGTTATT |  |
| AG9392_28_28 | GCACTGGGCAGAAATCACATCGCGTCAACACCCGCCGCGGGCCTTCGCGA |  |
| AG9517_12_18 | TTCCTTTTGATCGTGGTGATTTAGAGGGTGAACTCACTGGAACGGGGATG |  |
| AG9415_28_51 | GCCACTCCGGATTCGGGGATCTGAACCCGACTCCCTTTCGATCGGCCGAG |  |
| AG9424_28_60 | CACTCTCGACTGCCGGCGACGGCCGGGTATGGGCCCGACGCTCCAGCGCC |  |
| AG9343_18_17 | CTACGACGGTATCTGATCGTCTTCGAACCTCCGACTTTCGTTCTTGATTA |  |
| AG9334_18_8 | GCAGCCCCGGACATCTAAGGGCATCACAGACCTGTTATTGCTCAATCTCG |  |
| AG9467_5.8_2 | GCGTTCGAAGTGTCGATGATCAATGTGTCCTGCAATTCACATTAATTCTC |  |
| AG9433_28_69 | GATGGTTCGATTAGTCTTTCGCCCCTATACCCAGGTCGGACGACCGATTT |  |
| AG9391_28_27 | TCCCGCCGTTTACCCGCGCTTCATTGAATTTCTTCACTTTGACATTCAGA |  |
| AG9478_16_10 | GACCTGTGGGTTTGTTAGGTACTGTTTGCATTAATAAATTAAAGCTCCAT |  |
| AG9339_18_13 | GCCGGGTGAGGTTTCCCGTGTTGAGTCAAATTAAGCCGCAGGCTCCACTC |  |
| AG9496_16_28 | CAAAGTTATTTCTAGTTAATTCATTATGCAGAAGGTATAGGGGTTAGTCC |  |
| AG9515_12_16 | TAAACTTTCGTTTATTGCTAAAGGTTAATCACTGCTGTTTCCCGTGGG |  |
| AG9474_16_6 | AACCCTATTGTTGATATGGACTCTAGAATAGGATTGCGCTGTTATCCCTA |  |
| AG9393_28_29 | TGCTTTGTTTTAATTAAACAGTCGGATTCCCCTGGTCCGCACCAGTTCTA |  |
| AG9387_28_23 | CCTCTCATGTCTCTTCACCGTGCCAGACTAGAGTCAAGCTCAACAGGGTC |  |
| AG9417_28_53 | CAGGACCGACTGACCCATGTTCAACTGCTGTTCACATGGAACCCTTCTCC |  |
| AG9331_18_5 | CAAGCTTATGACCCGCACTTACTCGGGAATTCCCTCGTTCATGGGGAATA |  |
| AG9468_5.8_3 | GCAGCTAGCTGCGTTCTTCATCGACGCACGAGCCGAGTGATCCACCGCTA |  |
| AG9427_28_63 | CACCTTTTCTGGGGTCTGATGAGCGTCGGCATCGGGCGCCTTAACCCGGC |  |
| AG9342_18_16 | CATGGGAATAACGCCGCCGCATCGCCGGTCGGCATCGTTTATGGTCGGAA |  |
| AG9426_28_62 | TAGCGGATTCCGACTTCCATGGCCACCGTCCTGCTGTCTATATCAACCAA |  |
| AG9376_28_12 | CCTATTAGTGGGTGAACAATCCAACGCTTGGCGAATTCTGCTTCACAATG |  |
| AG9442_28_78 | CGCCCGGGGCGCACTGGGGACAGTCCGCCCCGCCCCCCGACCCGCGCGCG |  |
| AG9472_16_4 | CGGTCTGAACTCAGATCACGTAGGACTTTAATCGTTGAACAAACGAACCT |  |
| AG9519_5_1 | AAAGCCTACAGCACCCGGTATTCCCAGGCGGTCTCCCATCCAAGTACTAA |  |
| AG9488_16_20 | ATAGATTGGTCCAATTGGGTGTGAGGAGTTCAGTTATATGTTTGGGATTT |  |
| AG9379_28_15 | GCTTAAAACCCAAAAGGTCAGAAGGATCGTGAGGCCCCGCTTTCACGGTC |  |
| AG9411_28_47 | GATTTTCAAGGGCCAGCGAGAGCTCACCGGACGCCGCCGGAACCGCGACG |  |
| AG9511_12_12 | TGTGTTCAGATATGTTAAAGCCACTTTCGTAGTCTATTTTGTGTCAACTG |  |
| AG9371_28_7 | GCTATCCGAGGCCAACCGAGGCTCCGCGGCGCTGCCGTATCGTTCGCCTG |  |
| AG9491_16_23 | CCTAGTGTCCAAAGAGCTGTTCCTCTTTGGACTAACAGTTAAATTTACAA |  |
| AG9485_16_17 | TGGGTTGACAGTGAGGGTAATAATGACTTGTTGGTTGATTGTAGATATTG |  |
| AG9375_28_11 | GTAGGGTAAAACTAACCTGTCTCACGACGGTCTAAACCCAGCTCACGTTC |  |
| AG9423_28_59 | CGACGCACACCACACGCGCGCGCGCGCGCGCCGCCCCCGCCGCTCCCGTC |  |
| AG9505_12_6 | CCACCTCATGGGCTACACCTTGACCTAACGTCTTTACGTGGGTACTTGCG |  |
| AG9510_12_11 | AGGGCTAAGCATAGTGGGGTATCTAATCCCAGTTTGGGTCTTAGCTATTG |  |
| AG9347_18_21 | ATTCCATTATTCCTAGCTGCGGTATCCAGGCGGCTCGGGCCTGCTTTGAA |  |
| AG9378_28_14 | TTGGCCGCCACAAGCCAGTTATCCCTGTGGTAACTTTTCTGACACCTCCT |  |
| AG9429_28_65 | GGCACTCGCATTCCACGCCCGGCTCCACGCCAGCGAGCCGGGCTTCTTAC |  |
| AG9494_16_26 | ATAAATCTTCCCACTATTTTGCTACATAGACGGGTGTGCTCTTTTAGCTG |  |
| AG9338_18_12 | CCACGGAATCGAGAAAGAGCTATCAATCTGTCAATCCTGTCCGTGTCCGG |  |
| AG9453_28_89 | CGCCGACCCCACCCCCGGCCCCGCCCGCCCACCCCCGCACCCGCCGGAGC |  |
| AG9500_12_1 | GTTCGTCCAAGTGCACTTTCCAGTACACTTACCATGTTACGACTTGTCTC |  |
| AG9388_28_24 | TTCTTTCCCCGCTGATTCCGCCAAGCCCGTTCCCTTGGCTGTGGTTTCGC |  |
| AG9465_28_101 | GACTAATATGCTTAAATTCAGCGGGTCGCCACGTCTGATCTGAGGTCGCG |  |
| AG9481_16_13 | TACAGGTCCCTATTTAAGGAACAAGTGATTATGCTACCTTTGCACGGTTA |  |
| AG9476_16_8 | TCGCTTTGACTGGTGAAGTCTTAGCATGTACTGCTCGGAGGTTGGGTTCT |  |
| AG9390_28_26 | CACTAATTAGATGACGAGGCATTTGGCTACCTTAAGAGAGTCATAGTTAC |  |
| AG9410_28_46 | TGCTGCGGATATGGGTACGGCCCGGCGCGAGATTTACACCCTCTCCCCCG |  |
| AG9403_28_39 | CGGCCCCTGCCGCCCCGACCCTTCTCCCCCCGCCGCGCCCCCACGCGGCG |  |
| AG9435_28_71 | CTGCCCAGGCATAGTTCACCATCTTTCGGGTCCTAACACGTGCGCTCGTG |  |
| AG9516_12_17 | TGTGGCTAGGCTAAGCGTTTTGAGCTGCATTGCTGCGTGCTTGATGCTTG |  |
| AG9473_16_5 | TTAATAGCGGCTGCACCATCGGGATGTCCTGATCCAACATCGAGGTCGTA |  |
| AG9340_18_14 | CTGGTGGTGCCCTTCCGTCAATTCCTTTAAGTTTCAGCTTTGCAACCATA |  |
| AG9477_16_9 | GCTCCGAGGTCGCCCCAACCGAAATTTTTAATGCAGGTTTGGTAGTTTAG |  |
| AG9504_12_5 | CATAAGGGCTATCGTAGTTTTCTGGGGTAGAAAATGTAGCCCATTTCTTG |  |
| AG9483_16_15 | ACTGGTGATGCTAGAGGTGATGTTTTTGGTAAACAGGCGGGGTAAGATTT |  |
| AG9492_16_24 | GGGATTTAGAGGGTTCTGTGGGCAAATTTAAAGTTGAACTAAGATTCTA |  |
| AG9458_28_94 | CAAAGTTCTTTTCAACTTTCCCTTACGGTACTTGTTGACTATCGGTCTCG |  |
| AG9463_28_99 | CCGCGGGGCGGGGATTCGGCGCTGGGCTCTTCCCTGTTCACTCGCCGTTA |  |
| AG9380_28_16 | TGTATTCGTACTGAAAATCAAGATCAAGCGAGCTTTTGCCCTTCTGCTCC |  |
| AG9495_16_27 | TTCTTAGGTAGCTCGTCTGGTTTCGGGGGTCTTAGCTTTGGCTCTCCTTG |  |
| AG9497_16_29 | TTGCTATATTATGCTTGGTTATAATTTTTCATCTTTCCCTTGCGGTACTA |  |
| AG9484_16_16 | GCCGAGTTCCTTTTACTTTTTTTAACCTTTCCTTATGAGCATGCCTGTGT |  |
| AG9419_28_55 | CTGCACCTGCGGCGGCTCCACCCGGGCCCGCGCCCTAGGCTTCAAGGCTC |  |

| 2OM_r2r_1087 | rUrArCrCrCrUrUrCrArGrGrArArCrArArArUrArGrGrArUrGrGrArUrGmArCrArCrArUrArArUrCrCrArCrCrUrArUrCrCrCrArGrUrArGrGrArGrA | Oligonucleotides synthesized to test Nanopore 2'-O-Me calling. |
| --- | --- | --- |
| 2OM_r2r_1424 | rGrGrGrArCrCrCrGrGrCrCrArUrArArArGrCrArArGrArGrUrUrUmUrGrGrCrUrGrArArGrCrArArUrGrArGrCrCrArArGrUrArArCrArArArU |  |
| 2OM_r2r_8404_8408 | rCrUrGrUrArArGrGrGrArArArGrArArUrGrArGrArCrGrArGrCrUrGmArGrCrCmArGrCrArGrCrArGrArUrGrGrGrGrUrGrGrGrArGrCrArGrUrArUrC |  |
| 2OM_r2r_8960 | rCrGrGrArGrUrArCrUrUrCrArArGrArArCrUrGrCrUrGrArCrArUrCrGmArGrCrUrUrGrCrUrArCrArArGrGrGrArCrUrUrUrCrCrGrCrUrGrGrG |  |

# **Supplementary Table S2. Significant m6A modification sites in the HIV-1 sense epitranscriptome.**

# **Supplementary Table S3. Significant m5C modification sites in the HIV-1 sense epitranscriptome.**

# **Supplementary Table S4. Significant inosine modification sites in the HIV-1 sense epitranscriptome.**

# **Supplementary Table S5. Significant 2'-O-methylation modification sites in the HIV-1 sense epitranscriptome.**

# **Supplementary Table S6. Significant pseudouridine modification sites in HIV-1 sense epitranscriptome (low threshold).**

# **Supplementary Table S7. Significant differential modifications in transcript splice isoforms.**

# **Supplementary Table S8. Modifications that fall within a known splicing regulatory element.**

# **Supplementary Table S9. Significant modification sites in the NL4-3-GFP HIV-1 antisense epitranscriptome.**

# **Supplementary Table S10. Comparison of modification frequencies between new (v5.2.0) and old (v5.1.0) Oxford Nanopore Technologies modification-calling algorithms.**
